# Metagenomic Analysis of the Indian Ocean Picocyanobacterial Community: Structure, Potential Function and Evolution

**DOI:** 10.1371/journal.pone.0155757

**Published:** 2016-05-19

**Authors:** Beatriz Díez, Johan A. A. Nylander, Karolina Ininbergs, Christopher L. Dupont, Andrew E. Allen, Shibu Yooseph, Douglas B. Rusch, Birgitta Bergman

**Affiliations:** 1 Department of Molecular Genetic and Microbiology, Faculty of Biological Sciences, Pontificia Universidad Católica de Chile, Alameda 340, Casilla 114-D, C.P. 651 3677, Santiago, Chile; 2 Science for Life Laboratory, Department of Ecology, Environment and Plant Sciences, Stockholm University, Box 1031, 171 21 Solna, Sweden; 3 Center for Climate Change and Resilience Research (CR)2, Santiago, Chile; 4 BILS/Department of Bioinformatics and Genetics, Swedish Museum of Natural History, Box 50003, SE-10405, Stockholm, Sweden; 5 Microbial and Environmental Genomics, J. Craig Venter Institute, San Diego, CA 92037, United States of America; 6 Integrative Oceanography Division, Scripps Institution of Oceanography, University of California San Diego, La Jolla, CA 92037, United States of America; 7 Informatics Group, J. Craig Venter Institute, San Diego, CA 92037, United States of America; 8 Informatics Group, J. Craig Venter Institute, Rockville, MD 20850, United States of America; 9 Center for Genomics and Bioinformatics, Indiana University, Bloomington, IN 47401, United States of America; CSIR-National Institute of Oceanography, INDIA

## Abstract

Unicellular cyanobacteria are ubiquitous photoautotrophic microbes that contribute substantially to global primary production. Picocyanobacteria such as *Synechococcus* and *Prochlorococcus* depend on chlorophyll a-binding protein complexes to capture light energy. In addition, *Synechococcus* has accessory pigments organized into phycobilisomes, and *Prochlorococcus* contains chlorophyll *b*. Across a surface water transect spanning the sparsely studied tropical Indian Ocean, we examined *Synechococcus* and *Prochlorococcus* occurrence, taxonomy and habitat preference in an evolutionary context. Shotgun sequencing of size fractionated microbial communities from 0.1 μm to 20 μm and subsequent phylogenetic analysis indicated that cyanobacteria account for up to 15% of annotated reads, with the genera *Prochlorococcus* and *Synechococcus* comprising 90% of the cyanobacterial reads, even in the largest size fraction (3.0–20 mm). Phylogenetic analyses of cyanobacterial light-harvesting genes (chl-binding *pcb/isi*A, allophycocyanin (*apc*AB), phycocyanin (*cpc*AB) and phycoerythin (*cpe*AB)) mostly identified picocyanobacteria clades comprised of overlapping sequences obtained from Indian Ocean, Atlantic and/or Pacific Oceans samples. Habitat reconstructions coupled with phylogenetic analysis of the Indian Ocean samples suggested that large *Synechococcus*-like ancestors in coastal waters expanded their ecological niche towards open oligotrophic waters in the Indian Ocean through lineage diversification and associated streamlining of genomes (*e*.*g*. loss of phycobilisomes and acquisition of Chl *b*); resulting in contemporary small celled *Prochlorococcus*. Comparative metagenomic analysis with picocyanobacteria populations in other oceans suggests that this evolutionary scenario may be globally important.

## Introduction

The Indian Ocean is a highly dynamic tropical water body characterized by unique biophysical properties that strongly influence the diversity and performance of its biota [1, 2]. It is arguably the least studied of the oceans although it covers vast areas of the globe (~20% of the oceans; average depth of 3,700 m). The Indian Ocean is the warmest ocean and is currently vulnerable to rapid warming and increasing anthropogenic stressors, with unforeseen consequences.

The equatorial Indian Ocean is open to exchange with the Pacific Ocean by the “Indonesian Through Flow”, which allows large volumes of water and nutrients to enter, while it is enclosed by continental landmasses in the west (Africa) and the north (Arabia and India). The more accessible northern water masses are the most well studied parts, and encompass the Arabian Sea, the Red Sea, the Persian Gulf and the Bay of Bengal. These seas are highly influenced by the pronounced cross-equatorial Somali Current in the north-west (Indian Ocean equivalent to the Gulf Stream), which in turn is subject to the monsoonal reversals; as well as by water-run-off from bordering landmasses and rivers. The tropical equatorial open water regimes of the Indian Ocean are also subject to equatorial jets and under-currents. Satellite images (SeaWifs) indicate low chlorophyll concentrations in these waters in contrast to those of the macronutrient and iron-replete Arabian Sea and Bay of Bengal, which commonly exhibit high chlorophyll concentrations (summer monsoon) [3].

Morphological and genetic data, related to microbial communities, retrieved during the ‘pre-metagenomic’ era, primarily covering the Arabian Sea and coastal Indian Ocean regions, suggested a rich diversity of cyanobacteria. These range from massive and ecologically important ‘blooms’ of the diazotrophic genus *Trichodesmium*, covering up to hundred square kilometers of surface waters [4, 5], to benthic cyanobacterial mats or biofilms in coastal lagoons [6] and on coral reefs [7], to cyanobacterial epiphytes on seagrasses [8] and diazotrophic endosymbionts (*Richelia* spp.) in diatoms also known to bloom in the region [9]. As many of these cyanobacteria fix atmospheric dinitrogen, members of this phylum constitutes a valuable source of fixed nitrogen N (and carbon) in the Indian Ocean.

Tropical photoautotrophic marine picoplankton communities are typically dominated by representatives of the small celled unicellular cyanobacterial genera *Synechococcus* and *Prochlorococcus*, composed of diverse strains or ecotypes [10]. Through their wide distribution and high abundance, these genera represent the most common cyanobacteria in oceans, and are particularly evident in metagenomic data sets covering a range of ocean regions [11–13]. Comparative analysis of the distribution of *Synechococcus* and *Prochlorococcus* genotypes has suggested the occurrence of two complementary survival strategies, thought to be driven by nutrient availability [12], typified by quantitative dominance of small and slow-growing picocyanobacteria of the *Prochlorococcus* genus, with reduced genomes, over less abundant larger picocyanobacteria of the *Synechococcus* genus with more flexible (larger) genomes [10, 14, 15].

While numerous sequenced genomes are currently available for cultivated representatives of marine cyanobacteria, metagenomic surveys continue to identify new uncultivated lineages, further connecting genome content to ecological niche at finer scales (e.g. within *Prochlorococcus*). For example, new clades of high-light (HL) adapted *Prochlorococcus* clades were recently identified and characterized from high-nutrient, low-chlorophyll (HNLC), iron-depleted waters in the Pacific and Indian Oceans [16–18]. Reconstruction of consensus genomes of these *Prochlorococcus* ecotypes verified various hallmarks of genome streamlining. For example reduction in the number of Fe-binding proteins is thought to adaptation to chronically low levels of dissolved Fe [17].

Light harvesting genes are coupled to photosynthesis in all phototrophic organisms and therefore potentially represent useful molecular markers for picocyanobacteria in marine ecosystems. Proteins encoded by these genes are coupled to light-based pigment-dependent bioenergy capture in cyanobacteria and are composed primarily of chlorophyll (Chl) *a/b*-binding proteins (*pcb/isi*A light-harvesting gene family), the primary antenna of photosystem I and II (PSI and II), and cyanobacteria specific accessory pigments, found within thylakoid-associated phycobilisome complexes (phycobiliproteins; primary antenna of PSII). The phycobilisomes are composed of phycobiliproteins with different chromophores (phycoerythrin; phycocyanin and allophycocyanin). The phycobilisome complex has been shown to have a dynamic evolutionary history and display higher rates of molecular divergence relative to the more conserved ribosomal 16S rRNA gene. As a result phycobiliprotein genes have become useful genetic markers for microdiversity analyses (see e.g. [14, 19]. *Synechococcus* light-harvesting complexes genes exhibit the canonical structural components found in most other cyanobacteria, while *Prochlorococcus* harbors both Chl *a*2 and *b*2 and lacks phycobilisomes, although some strains may contain the gene encoding phycoerythrin (*cpe*B) [14, 20]. In *Synechococcus*, the light-harvesting Chl *a*-binding protein (CP43-like Chl *a*-binding protein) is encoded by the *isi*A gene. Other than binding Chl *a*, the IsiA protein also has a role is dissipation of light energy and protects PSI from excessive excitation under iron-depletion [21]. In *Prochlorococcus*, the Chl *a/b*-binding proteins (CP43-like Chl-binding proteins) are encoded by at least eight different *pcb*-like genes (*pcb*A to *pcb*H) and are the principal light-harvesting genes [22–23].

Metagenomic approaches are well suited expand knowledge and comprehension of the diversity, ecology, evolution and functional potential of the natural marine microbial world [11, 24, 25]. Using such approaches we characterized the microbial community in equatorial open waters of the Indian Ocean, with focus on photoautotrophic picocyanobacteria. Metagenomic analyses were based on microbial samples collected along a transect (18 Stations) stretching from The Indonesian Through flow in the east towards coastal areas off the coast of Tanzania and southwards outside Madagascar in the west (approx. 10°S to 20°S). The marine picocyanobacterial genera *Prochlorococcus* and *Synechococcus* were specifically targeted and their genetic biogeography was determined using the phylogeny of light-harvesting protein systems. Analyses of our metagenomic dataset suggested a community that contains mostly clades present in several locations of the Pacific and Atlantic Oceans (GOSI stations), but also other clades, which appear to be restricted to the Indian Ocean or to the Indian Ocean plus one of the other two basins (Atlantic or Pacific). Shifts in ecological distribution related to local conditions and underlying picocyanobacteria biogeography are also apparent. Habitat reconstruction analyses indicate the ancestral nature of *Synechococcus* coastal strains and suggest that they are the progenitors of the small and dominant open ocean *Prochlorococcus* spp. dominate contemporary oceans.

## Material and Methods

### Sampling and metadata

**Fig 1:**
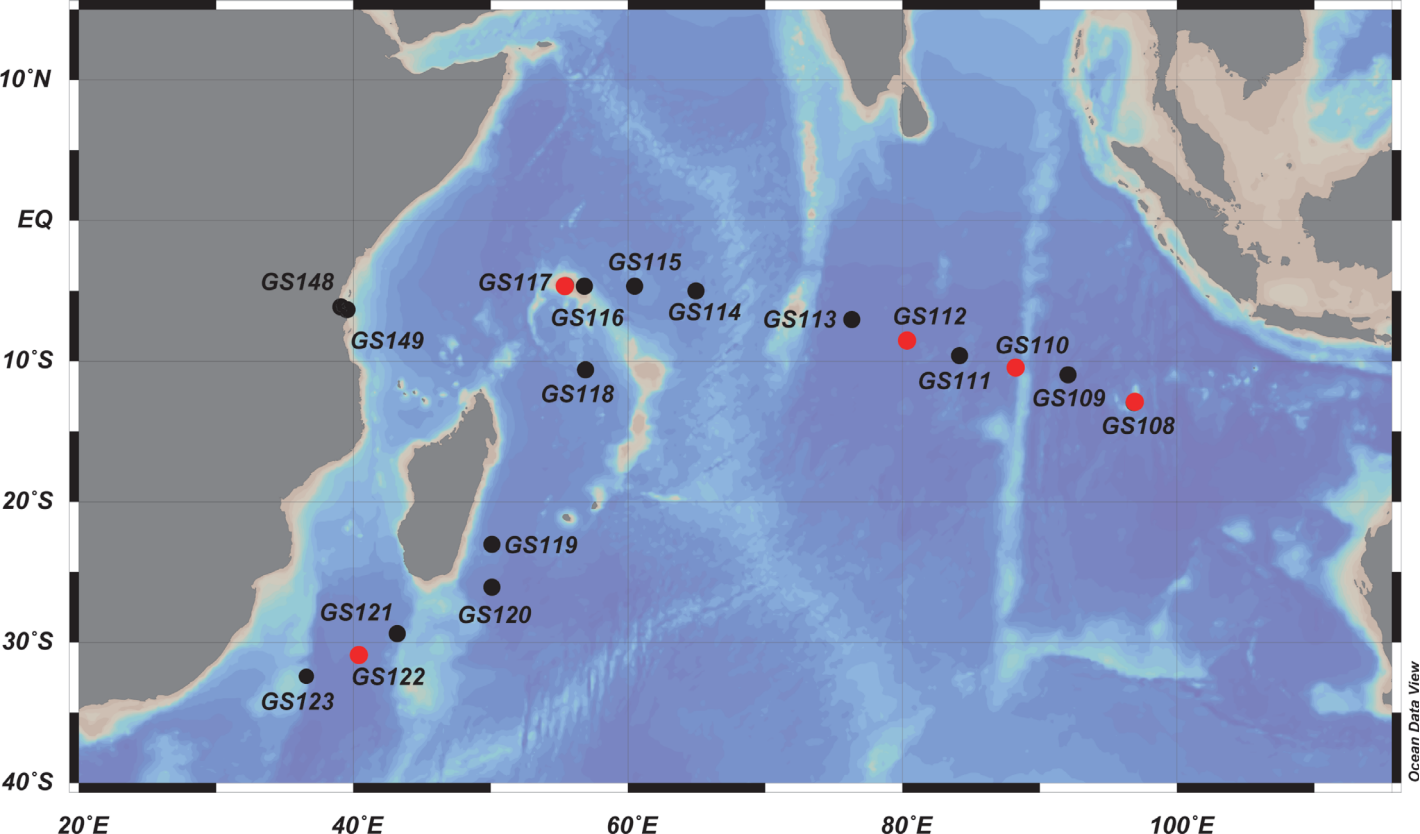
Map of Indian Ocean indicating metagenomic sampling stations along the east-west equatorial transect in GOSII.

Sampling and metadata at each location were previously described by Rusch et al. and Williamson et al. [17, 26]. S1 Table contains a summary of the metadata from all the 18 Indian Ocean samples investigated in the present study. Briefly, surface water samples (400 L) were collected from 16 sites from the tropical Indian Ocean between August and October 2005 aboard the *S/V Sorcerer II*. Two additional sites were sampled off the island of Zanzibar, Tanzania using alternate vessels (Fig 1; S1 Table). Permission for sampling in territorial waters was obtained from the following authorities: GS148 and GS149 (Tanzania): Director of Co-ordination, Chief Minister’s Office, Revolutionary Government of Zanzibar (permission received September, 2005); GOS108 Cocos Keeling Islands (territory of Australia National Biodiversity Section): Department of the Environment, Commonwealth of Australia (agreement signed November 4, 2004); GS116 and GS117 (Seychelles): Principal Secretary, Nature and Conservation, Ministry of Environment and Natural Resources, Republic of Seychelles (agreement signed August 18, 2005); GS120 (Madagascar): National Ministry of Education and Scientific Research, Republic of Madagascar (permission received September 8, 2005). The rest of the samples were obtained from International Waters and not requiring sampling permits. The microbial community was prefiltered using a 20 μm mesh Nytex net (General Oceanics) and size fractionated by serial filtration through 3.0, 0.8, and 0.1 μm membrane filters (Millipore). Iron concentrations were obtained by simulation using the ocean biogeochemical element cycling (BEC) model [17]. In addition, nitrate and phosphate monthly averages were derived from the NODC World Ocean Atlas [27]. Additional sequences from several Atlantic and Pacific Ocean samples from GOSI (S1 Table) were also included in the present study for comparative purposes.

### Metagenomes sequencing and post-processing of data

Methods describing DNA extraction from filters, construction of clone libraries, template preparation, and automated cycle sequencing can be found in Rusch et al. [25]. In addition to Sanger libraries, 454 Titanium libraries were prepared from amplified bacterial from samples for selected stations (see below) and pyrosequenced at JCVI as described in Dupont et al. [28]. While the microbes retained within the 0.1–0.8 μm size fraction were sequenced from all stations, the Indian Ocean stations GS108, GS110, GS112, GS117 and GS122 were selected for comprehensive metagenomic exploration with sequencing performed on all microbial size fractions (0.1–20 μm). Read proportions of *Prochlorococcus* and *Synechococcus* from the five Indian Ocean selected stations were calculated both within the cyanobacterial population and within the total community (all annotated reads). Read abundances for functional genes were retrieved from METAREP [29] and normalized to all sequenced reads or to *rec*A for each cyanobacterial genera respectively.

Metagenomic reads were annotated functionally (gene name, gene symbol, GO terms, EC numbers, Kegg Ortholog (KO) and JCVI functional role categories) and taxonomically with the JCVI metagenomic annotation pipeline [30]. All metagenomes are available at iMicrobe (http://data.imicrobe.us/project/view/26) and at the European Nucleotide Archive (ENA) under project: PRJEB8968. Genes encoding Chl-binding proteins and phycobiliproteins were identified with the following KO annotations: Chl-binding proteins, KO8918 (PcbA), KO8918 (PcbB), KO8920 (PcbC), KO8921 (PcbD), KO8922 (PcbE), KO8923 (PcbF), KO8924 (PcbG), KO8925 (PcbH); Phycobiliproteins α subunits, KO2092, KO5376, KO2284, and β subunits, KO2093, KO2285, KO5377. For these light-harvesting genes, the taxonomic annotations were verified by considering the best BLAST hits (blastp of amino acid sequences against NCBI nr database) as tentative closest taxon (down to genus level). The subset of peptides analyzed in this study can be found here https://scripps.ucsd.edu/labs/aallen/data/.

### Picocyanobacterial light-harvesting genes phylogenetic analysis and habitat reconstruction

Peptide sequences from annotated open reading frames corresponding to light-harvesting genes were quality checked for stop codons, and aligned using the software MUSCLE v.3.8 [31] together with homologous sequences taken from Genbank (S2 Table). A best-fit amino acid model was selected using ProtTest v.3 [32], followed by maximum likelihood inference of phylogeny using the software PhyMl, v.3 [33]. Where possible, the trees were rooted using one of the reference sequences based on prior information related to cyanobacterial phylogeny (e.g., [15]). Phylogenetic trees were then analyzed with the AdaptML software v.1.0 [34]. Indian Ocean geographic location defined by stations, size fraction defined by 20–3 μm, 3–0.8 μm and 0.8–0.1 μm, and an operational definition of coastal (< 200 m depth) and open ocean (>200 m depth) were used for subsequent habitat reconstruction stepping backwards through past evolutionary events by AdaptML. AdaptML uses sample ecology information to identify genetically- and ecologically-distinct populations. Correlations between each light-harvesting gene/subunit sequence within the Indian Ocean (GOSI)-GOSII with taxonomy, cell size, and location including other GOSI stations along different regions on the Pacific and Atlantic basins were visualized and investigated using tools at Interactive Tree of Life (http://iTol.embl.de, [35]).

To test for differences in genetic diversity within and among sample areas (Indian Ocean, Atlantic and Pacific), we performed tests of analysis of molecular variance (AMOVA) and homogeneity of molecular variance (HOMOVA) [36] as implemented in MOTHUR, v.1.33.3 [37], based on the JTT-sequence distances calculated in PROTDIST [38].

## Results

The locations of the 18 sampling stations along the Indian Ocean longitudinal transect (12°S, 96°E to 4°S, 39°E) are shown in Fig 1. The 0.1–0.8 μm fraction was sequenced for all samples while five Indian Ocean samples (GS108, GS110, GS112, GS117 and GS122) were examined in greater detail by additionally sequencing the 0.8–3.0 and 3.0–20.0 μm size fractions.

Temperature and salinity values ranged from 20 to 28°C and 32 to 35 psu respectively (S1 Table). Chlorophyll *a* levels ranged from 0.1–0.2 mg/m^3^, indicating that truly oligotrophic conditions prevailed. Maximum Chl *a* levels (0.45 mg/m^3^) were observed in the more coastal stations GS117 (close to the islands of the Seychelles) and GS148 and GS149 (off the island Zanzibar, Tanzania).

Metadata for the Atlantic and Pacific Ocean stations used here for comparison to the Indian Ocean are also presented in S1 Table. Briefly, metadata of the Atlantic Ocean stations in the Sargasso Sea and Caribbean Sea were more similar to those of the Indian Ocean, while lower temperature and salinity and a higher total Chl *a* were more characteristic of the North American East coast. In general, the Pacific Ocean stations (Eastern Tropical Pacific, Galapagos Island, Tropical South Pacific and Polynesia Archipelagos) were similar to the Indian Ocean in terms of temperature and salinity, while Chl *a* was higher in Galapagos waters.

### Indian Ocean cyanobacterial occurrence and distribution

**Fig 2:**
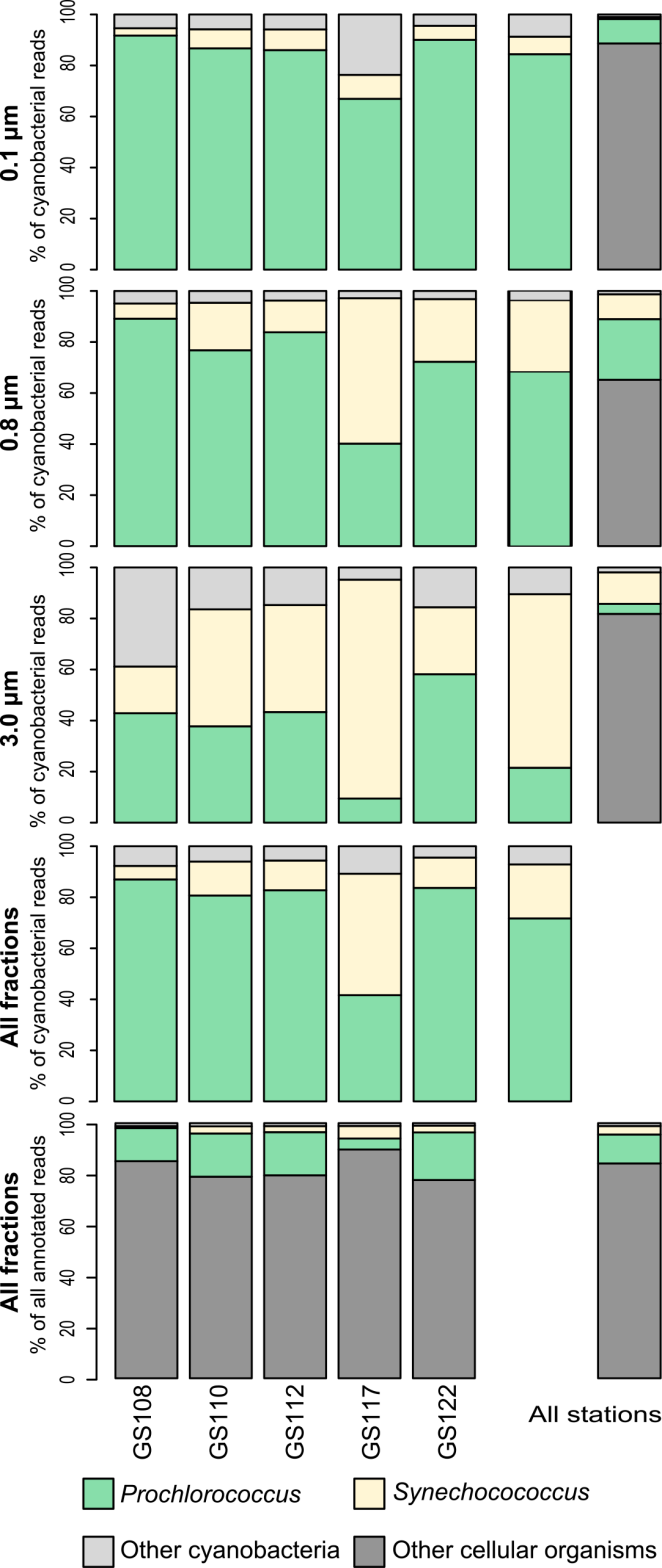
Proportionate abundances of the picocyanobacteria *Prochlorococcus* and *Synechococcus* among cyanobacteria and other microorganisms in the Indian Ocean sorted by size fractionation. The total number of genes taxonomically referring to picocyanobacteria and other microbes are given per size fraction at the Indian Ocean stations GS108, GS110, GS112, GS117 and GS122.

A total of 3,079,311 DNA sequences where retrieved from the Indian Ocean metagenomic data set of which 2,261,836 were annotated (including archaea and eukaryotes referred to as “other cellular organisms’) (Fig 2). Overall, a total of 349,892 cyanobacterial sequences were related to the unicellular genera *Prochlorococcus* and *Synechococcus* and constituted a significant fraction (from 10 to 20%) of reads for the total Indian Ocean microbial community (including all stations and size fractions; the latter subsequently denoted as 0.1, 0.8 and 3.0 μm size fractions). The relative proportion of these picocyanobacterial reads varied, as a fraction of total reads across all size classes, between 9% (GS117) and 21% (GS122). Together these picocyanobacteria also dominated the cyanobacterial reads in all size fractions, comprising 91% (0.1 μm), 96% (0.8 μm) and 90% (3.0 μm), respectively. *Prochlorococcus* reads alone made up 75% of the total cyanobacterial reads, while *Synechococcus* represented 20%, except at the coastal station GS117 (close to the islands of Seychelles), where *Synechococcus* dominated. Such dominance was also evident at two additional coastal stations, GS148 and GS149 off the island of Zanzibar (Tanzania). Larger cyanobacteria (size fraction >3.0 μm) accounted for less than 10% of the total cyanobacterial community on a per read basis. However, in the largest size fraction at a station located in an eastern Indian Ocean reef area (GS108) defined as ‘coastal’ (see M&M), reads related to the large filamentous non-heterocyst genus *Trichodesmium* were prominent suggesting an ongoing bloom.

### Picocyanobacterial diversity inferred through light-harvesting gene analysis

Picocyanobacterial light-harvesting genes (Chl-binding and phycobiliprotein related genes) were detected at all 18 Indian Ocean stations and in the majority of the size fractions. A total number of 1,100 light-harvesting gene sequences were identified, out of which the majority (817) encoded for Chl-binding proteins (S1A Fig). In regard to phycobiliproteins, 120 sequences encoded for the *Synechococcus* α-subunits of allophycocyanin (*apc*A), phycocyanin (*cpc*A) and phycoerythin (*cpe*A); 245 sequences represented the β-subunits of allophycocyanin (*apc*B), phycocyanin (*cpc*B) and phycoerythin (*cpe*B) in *Synechococcus* and some phycoerythin (*cpe*B) sequences from *Prochlorococcus* (S1B and S1C Fig). The smallest size fraction (0.1 μm) of GS108 stands out in terms of picocyanobacterial light-harvesting gene abundance, and most Chl-binding sequences show best BLAST hits to *P*. *marinus* (S1A Fig). In contrast, at the coastal station GS117 the larger fractions showed the highest abundance of sequences with best BLAST hits to *Synechococcus* (S1B Fig). Sequences dominant at GS108 are virtually absent in GS117 and *vice versa*.

In terms of genetic diversity of light-harvesting genes from picocyanobacteria on a geographic scale, samples from Indian Ocean did not differ significantly from those in the Atlantic and Pacific Ocean stations from GOSI. For instance, HOMOVA analyses for testing genetic diversity in multiple sampled areas, did not identify significant differences for the phycobiliprotein α- and ß-subunit data sets (see S3 Table). On the other hand, the HOMOVA tests of the Chlorophyll-binding protein (Pcb/IsiA) data set clearly highlights the Atlantic sequences as being significantly different from the other compared areas. The AMOVA, which tests whether the genetic diversity within each sample area is significantly different from the genetic diversity of the pooled areas, identified some differences based on the phycobilisome ß-subunits (S4 Table). Again, sequences from the Atlantic Ocean, along with Galapagos, and Polynesian Archipelagos showed some genetic differences.

### *Prochlorococcus* light-harvesting genes—phylogenetic affiliation

**Fig 3:**
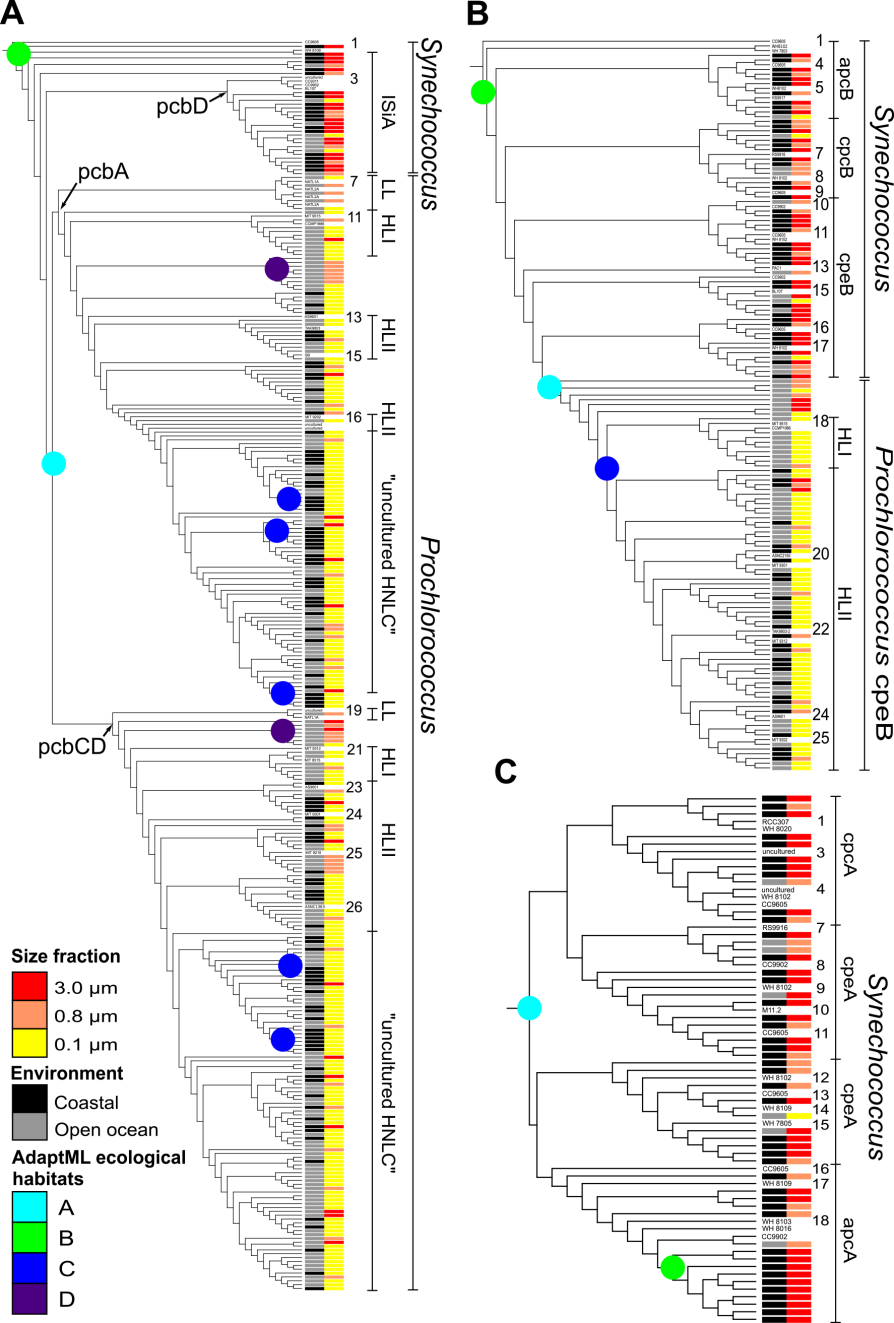
Phylogenetic reconstruction of picocyanobacteria and their habitat distribution along the Indian Ocean transect. Metagenomic samples from stations GS108, GS110, GS112, GS117 and GS122 were analyzed. Amino acid sequences (longer than 80 amino acids) from *Synechococcus* and *Prochlorococcus* were collected from the five stations, and aligned using MUSCLE [31] together with reference sequences taken from GenBank. The phylogenetic tree was inferred by maximum likelihood using PhyML and the VT+Γ model for sequence evolution [33]. AdaptML analysis [34] was used to map their characteristics and habitat predictions. The inferred habitats are identified by colored circles at internal nodes, and labeled A–D. Colored bars indicate Environment (Column no. 1): coastal (black), open ocean (gray), and Size fractions (Column no. 2): 0.1 μm (yellow), 0.8 μm (orange), 3.0 μm (red). (A) Pcb/IsiA, light-harvesting chlorophyll-binding peptides. (B) Light-harvesting phycobilisome ß-subunits (cpe, cpc, apc). (C) Light-harvesting phycobilisome α-subunits (cpe, cpc, apc). Sequences included as references are given in S2 Table, and numbered from 1 and downwards.

**Fig 4:**
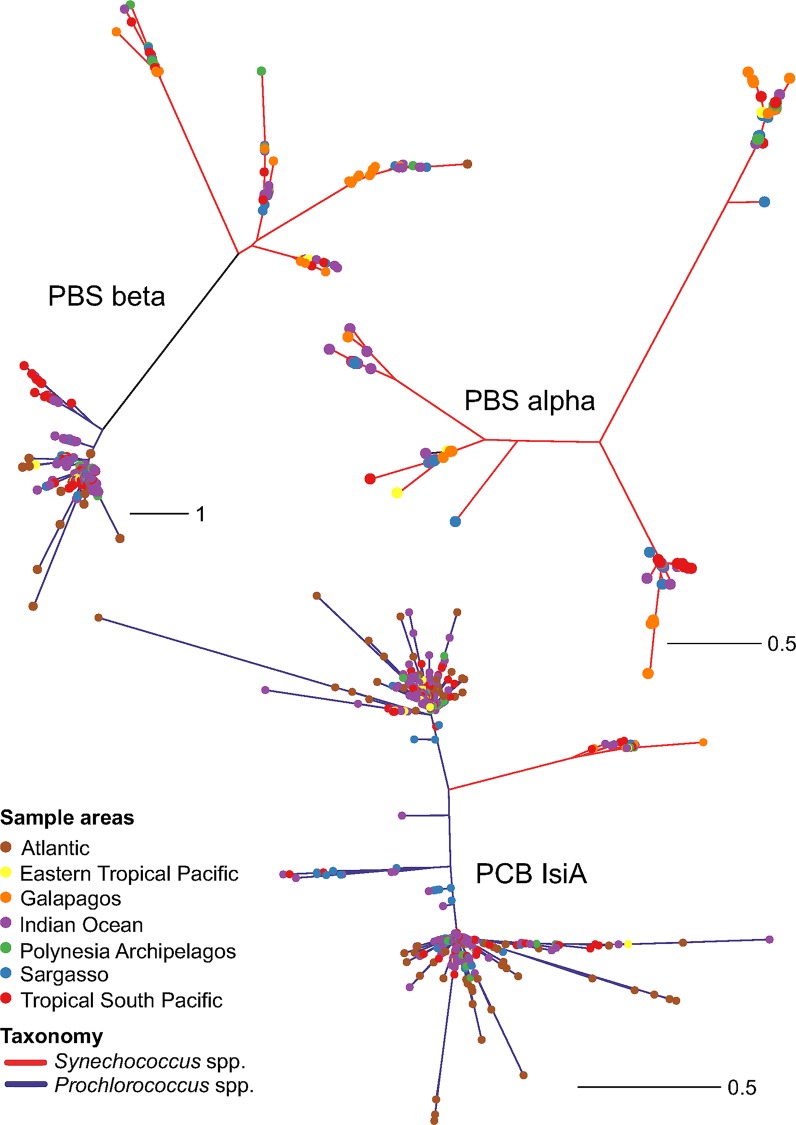
Unrooted picocyanobacterial gene trees inferred using maximum likelihood of sampled in the Indian Ocean (GOSII), and the Atlantic and Pacific Oceans (GOSI). Sampling locations (colored dots), and taxonomy (colored edges). (A) Pcb/IsiA, light-harvesting chlorophyll-binding peptides. (B) Light-harvesting phycobilisome ß-subunits (*cpe*, *cpc*, *apc*). (C) Light-harvesting phycobilisome α-subunits (*cpe*, *cpc*, *apc*). Scale bar indicates expected number of substitutions per site.

Phylogenetic analysis of the *Prochlorococcus* specific Chl *a/b*-binding protein genes (*pcb*A-H) clearly underlines their dominance in the smaller fractions at Indian Ocean stations (Fig 3A). High light (HL) adapted ‘group I’ strains of *Prochlorococcus* containing *pcb*CD genes (associated to PSI; [14]) dominated at all Indian Ocean stations; while low light (LL) adapted ‘group II’ strains containing *pcb* genes (associated to PSII; [14], e.g. *P*. *marinus* str. NATL1A, 2A) were restricted to one station, GS122. Moreover, most of the Indian Ocean *Prochlorococcus* sequences were distantly related to the HLII *Prochlorococcus marinus* spp. such as MIT9215, MIT9301, ASNC1363 and AS9601, indicating that these sequences may represent new clades of uncultured *Prochlorococcus* spp. (Fig 3A). Low numbers of *Prochlorococcus* reads were recovered from stations defined as coastal, such as stations GS117, GS148 and GS149. Furthermore, comparative analyses of the Indian Ocean sequences to other ocean geographical locations (North American East coast, Caribbean Sea, Sargasso Sea, Eastern Tropical Pacific, Tropical South Pacific, Polynesian Archipelagos, and Galapagos Island), illustrates the relatedness between most of these picocyanobacteria sequences (Fig 4A).

Overall, *pcb-*like phylotypes of *Prochlorococcus* in the Indian Ocean showed similarities to other sequences from GOSI stations in the Atlantic and the Pacific Oceans (Fig 4A; S2 Fig). In particular, a *pcb*B-H clade (S2 Fig) related to the LL cultured *Prochlorococcus* sp. PAC1 and MIT0801 were well represented in the Indian Ocean, as well as in the Sargasso Sea and Tropical South Pacific samples (generally open ocean stations). *pcb*A sequences were found at all locations, and a specific clade was formed by sequences from the Indian Ocean together with the Tropical South Pacific and Eastern Tropical Pacific. *pcb*D gene sequences were also present at all locations, while a large Indian Ocean clade was identified that also included sequences from the Atlantic Ocean, Tropical South Pacific, Eastern Tropical Pacific and Polynesian Islands. These sequences were most similar to the previously identified strain HLIV-HNLC2 *Prochlorococcus* sp. W2 [39]. As for *pcb*A, a specific *pcb*D clade was identified, with sequences restricted to only the Indian Ocean, Tropical South Pacific and Eastern Tropical Pacific samples.

Although *Prochlorococcus* lacks phycobilisomes [40], both HLI and HLII phylotypes were identified using phycoerythrin *cpe*B as the query (Fig 3B; S1C Fig). Congruent with the Chl-binding protein encoding genes, *cpe*B gene sequences clearly originated from small-celled *Prochlorococcus* and were particularly abundant in the smallest size fractions (Fig 3B; S1C Fig). These were mostly affiliated to HLII adapted *Prochlorococcus* strains (*e*.*g*. *P*. *marinus* AS9601, MIT9312, MIT9301, MIT 9202 and ASNC2150). A few *pcb* sequences at the station GS122 were affiliated to HLI adapted *Prochlorococcus marinus* strains CCMP1986 and MIT9515. A limited set of the *cpe*B gene clades from the Indian Ocean affiliated with *Prochlorococcus marinus* spp. HLII strains TAK9803 (RCC264, isolated from the Pacific Ocean), MIT 0604 and the HLIV-HNLC2 strain W2 [39], but were also observed at other GOSI locations (Fig 4B; S3 Fig).

### *Synechococcus* light-harvesting genes—phylogenetic affiliation

*Synechococcus* phylotypes identified by the *isi*A gene (group III; [14]) represent distinct components of the Indian Ocean picocyanobacterial community, specifically at stations defined as coastal and in the larger size fractions, i.e. at GS117 and at GS148-149 (3.0 μm size fraction) (Figs 3A and 4A). Phylotypes of *pcb*D were related to *Synechococcus* sp. Group I, cluster 5.1A (*e*.*g*. CC9902, WH8109, CC9311 and BL107), which includes strains able to adapt their light harvesting spectra via chromatic adaptation (sensu Palenik 2001) [41]. They *pcb*D gene sequences retrieved were also mostly related to *pcb*D-*isi*A gene sequences from the Galapagos Islands, Tropical South Pacific and Polynesian Archipelagos (Fig 4A; S2 Fig). In contrast to *Prochlorococcus* sequences the Indian Ocean *Synechococcus* sequences were distantly related to those from the Atlantic Ocean.

Sequences for the phycobilisome α-subunits (*cpe*A, *cpc*A and *apc*A), which are taxonomically exclusive for *Synechococcus*, also highlight the prevalence of this genus in coastal sites and in larger size fractions (Fig 3C). However, not all Indian Ocean α-subunit phylotypes correlated to ß-subunit phylotypes (Fig 3B and 3C). Rather, the α-subunits sequences were mostly found in the coastal stations. Examining the more conserved allophycocyanin subunit genes (*apc*AB) showed that several *apc*AB clades of *Synechococcus* exist among the Indian Ocean sequences and that these are affiliated to cultured representatives, such as *Synechococcus* strains CC9605 (belonging to the oligotrophic 5.1A group II), RS9917 and WH8102/03/09 (Fig 3B and 3C). The *apc*AB Indian Ocean sequences comprised clades with sequences from different locations in the Pacific Ocean (*e*.*g*. Galapagos Island, Tropical South Pacific and Polynesian Archipelagos), and the Sargasso Sea, but not with Atlantic coastal (*e*.*g*. North American East coast) or the coastal Eastern Tropical Pacific stations (Fig 4B and 4C; S3 and S4 Figs).

Marine *Synechococcus* strains can possess two different phycoerythrin (PE) proteins (PEI and PEII), representing two additional bilin pigments, phycourobilin (PUB) and phycoerythrobilin (PEB) that bind to the PE protein [40]. PEI contains lower levels of PUB and prefers red light. PEB may be converted to PUB thereby raising the PUB/PEB ratio [42], a process that underpins chromatic adaptation [41]. PE-rich *Synechococcus* phylotypes dominated the Indian Ocean transect and are affiliated with subcluster 5.1 (Fig 3B and 3C) and were related to the PEII PUB-rich (blue light) *Synechococcus* WH8102 and CC9605 group II and III (high PUB:PEB ratio), and to *Synechococcus* CC9902 and BL107 phylotypes. The PEI related *Synechococcus* strains primarily occurred at GS112 and GS117. Furthermore, PEII sequences (group II; [14]) in the Indian Ocean formed two or three clusters (depending of the subunit analyzed) (Fig 3B and 3C). Yet other clades containing *cpc*B and *cpe*B gene sequences (ß-subunits; Fig 3B) were common in the small size fraction of most Indian Ocean stations, and were related to *Synechococcus* spp. such as str. WH8102 (5.1 A III, oligotrophic areas) and many sequences from GOSI (Fig 4B; S3 Fig). The phycocyanin-rich *Synechococcus* phylotypes (*e*.*g*. RS9917) were less abundant overall, but prominent at the coastal stations GS117 (larger fractions), GS148 and GS149, although also observed at GS112 and GS122. These findings corroborate previous reported abundances at coastal Indian Ocean stations of *Synechococcus* CC9605 phylotypes using metagenomics data and associated fragment recruitment analyses [12]. Both *cpc*AB and *cpe*AB sequences from Indian Ocean *Synechococcus* were closely related to sequences from several other GOSI locations such as Sargasso Sea, Eastern and Southern Tropical Pacific, Galapagos and Polynesian Archipelagos, but distantly related to any sequence from the North American East coast here investigated, as also observed for the *apc*AB genes (Fig 4B and 4C; S3 and S4 Figs).

### Gene content related to energy metabolism in Indian Ocean picocyanobacteria

**Fig 5:**
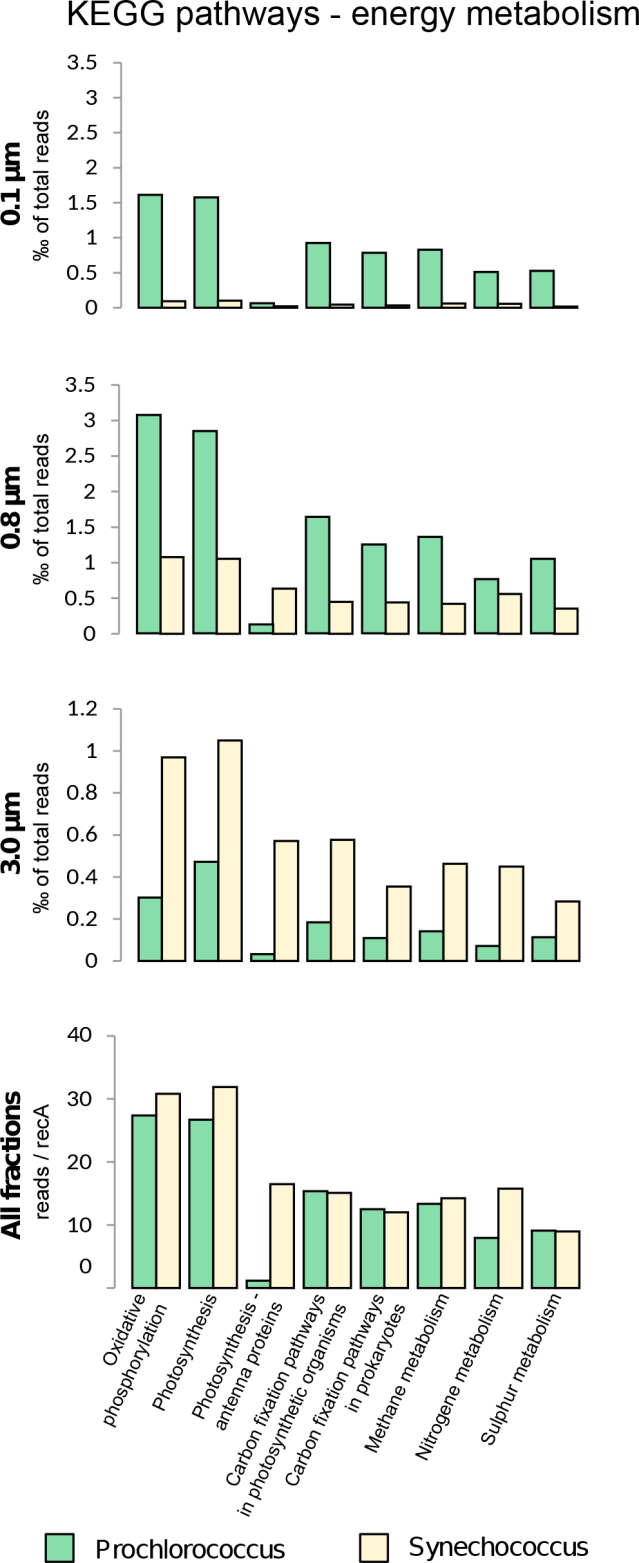
The distribution of ‘Energy metabolism’ related KEGG Orthology (KO) categories among picocyanobacteria in the Indian Ocean. KO categories and their relative abundances for picocyanobacteria are given per size fraction at the Indian Ocean stations GS108, GS110, GS112, GS117 and GS122. The last panel is showing number of reads in all fractions normalized to number of *rec*A for each respective genus.

To obtain insights into the metabolic potential and system behavior of the Indian Ocean picocyanobacteria, combined protein sequences annotated as either *Prochlorococcus* or *Synechococcus* were retrieved from five selected stations (GS108, GS110, GS112, GS117 and GS122) and classified according to the KEGG cellular pathways [43] and normalized to the *rec*A gene. Reads annotated as genes related to energy metabolism were specifically targeted, with the dominating sub-categories being ‘oxidative phosphorylation’ and ‘photosynthesis’- related pathways (Fig 5). The majority of the sequences were affiliated to *Prochlorococcus* at all stations and across all size fractions, with the exception of the two larger fractions at GS117, which were dominated by *Synechococcus* (S5 Fig). The *rbc*L gene (large subunit of ribulose-1,5-bisphosphate carboxylase/oxygenase) of the *Prochlorococcus* Calvin-Benson cycle dominated at all stations and size fractions except for the larger size fractions at GS117 where *Synechococcus rbc*L reads dominated (data not shown). As seen in Fig 5, the proportion of reads in energy related KEGG categories was similar between size fractions for both genera and with their relative abundance being highest in the medium size fraction (0.8–3.0μm), which was approximately three-fold higher than that of the largest fraction (Fig 5).

It is important to note that several KEGG categories overlap sharing several enzymes, e.g. the majority KOs classified here as belonging to the category ‘methane metabolism’ are also involved in glycolysis or other types of carbon metabolism and should therefore not be interpreted as an indication of methane metabolism in cyanobacteria.

Normalizing all *Synechococcus* and *Prochlorococcus* reads to *rec*A showed that *Synechococcus* dominated most categories, except those related to carbon fixation and sulfur metabolism. In particular, *Synechococcus* dominated the categories “antenna proteins” and “nitrogen metabolism”. The observed difference in nitrogen metabolism may be explained by an absence of *Prochlorococcus* ferredoxin-nitrate reductase (*nar*B) genes in our data set, while, *nar*B genes associated with *Synechococcus* were abundant at all stations (S6 Fig). Only a small percentage of *Prochlorococccus* populations contained phycoerythrin, that is in turn reflected as a large discrepancy between the two picocyanobacteria in the “antenna pigment” category (Fig 5). The *Prochlorocccus* reads within this category are mostly *cpe*B and *cpe*S.

The abundance of cyanobacterial genes related to stresses (nutrient and oxidative) was also examined, as high-light, low nutrient conditions are known to prevail in Indian Ocean surface waters. For instance sensor-response histidine kinase encoding genes, involved in signaling phycobiliprotein degradation under low nutrient conditions (*nbl*S) and high light stress (*hli*A), as well as genes for the oxidative stress related putative nickel-containing superoxide dismutase (NiSOD) [44] were found in high abundance at all Indian Ocean stations (S5 Fig). With the exception of the *Synechococcus nbl*S gene in the largest fraction, the majority of reads were associated with *Prochlorococcus*, and most abundant in the medium size fraction.

### Reconstruction of picocyanobacterial ecological habitat and evolutionary events

Ecological habitat reconstruction was performed using the picocyanobacterial light-harvesting genes from Indian Ocean stations GS108, GS110, GS112, GS117 and GS122 (Fig 3A–3C). We used sequence information to identify genetically- and ecologically-distinct picocyanobacterial populations, thereby identifying shifts in community 'ecological habitats'. The latter are defined here as a combination of ecological characters such as the size fractions of the community (0.1–0.8, 0.8–3 and 3.0–20 μm), geographical parameters (open ocean or coastal), and clade affinities combined with phylogenetic branch lengths (level of diversification). To our knowledge, this is the first time that this type of analysis has been performed for the marine picocyanobacteria. As seen in Fig 3A–3C, such reconstructions identified several distinct “ecological habitats' within each of the picocyanobacterial phylogenies. Independent of the target gene, we detected major shifts in ecological habitat corresponding to the phylogenetic split between *Synechococcus* and *Prochlorococcus* (Fig 3A and 3B). For instance, AdaptML analyses based on evolution of phycobiliprotein ß-subunits genes indicate that three ecological habitat shifts have occurred (green, light blue and dark blue clades) which correspond to changes in both picocyanobacterial cell size and environmental characteristics (Fig 3B). These data suggest that a large-celled picocyanobacterial ancestor population (0.8 and 3.0 μm size fractions, red, Fig 3B), which is most closely related to more coastal extant *Synechococcus* phylotypes (black, Fig 3), expanded their ecological niche towards more open ocean habitats (illustrated as grey). Such derived phylotypes are represented in GS112. Possibly the process of diversification was initiated within their original ecological habitat (dark blue), through loss of genes, such as those encoding most phycobiliproteins, except for some *cpe*B genes (Fig 3B); thereby streamlining their genome and decreasing in cell size, followed by a “reappearance” in the small size fraction (yellow) in habitats represented by for instance the stations GS108, GS110 and GS122. Putatively these evolutionary steps, in part, eventually gave rise to the picocyanobacteria we today identify as the genus *Prochlorococcus*, characterized by small cells and small genomes.

## Discussion

Based on targeting several fundamental light harvesting genes encoding proteins coupled to solar-based bioenergy production, from a comprehensive metagenomic data set from microbes in the Indian Ocean, we provide the first detailed overview of picocyanobacteria in this region. Not surprisingly, our data strongly support the ubiquity of picocyanobacteria in this understudied ocean and extend our knowledge of their phylogenetic diversity and biogeography beyond that of the more well characterized clades of the Atlantic and Pacific Oceans [45, 14].

We analyzed samples from surface waters across an equatorial longitudinal transect in the Indian Ocean with relatively homogeneous temperature, salinity, Chl *a* and nutrient concentrations (S1 Table). Indian Ocean surface waters are characterized by high temperatures and levels of radiation as well as oligotrophy, with low concentrations of macronutrients and iron [46]. Iron limitation is a consequence of high N:Fe ratios in upwelling currents and lack of nearby terrigenous or aeolian sources. In the southern tropical Indian Ocean region, austral winter Ekman pumping and westward propagating Rossby waves are the primary means of nutrient supply to the iron-limited waters [47]. In particular, iron limitation may prevail along the Indian Ocean transect examined here [17]. However, in coastal and shelf-influenced waters, iron levels are not thought to limit primary production [48]. Therefore, we hypothesized that the coastal waters at stations GS117 and GS108 (depths <200 m) would harbor picocyanobacteria with more versatile nutrient-acquisition strategies compared to the open water oligotrophic stations (depth >200 m).

Based on the phylogeny of picocyanobacterial phylotypes closely affiliated with cultured and uncultured *Prochlorococcus* and *Synechococcus*, the occurrence and distribution of ecotypes of these two genera across the entire equatorial Indian Ocean surface water segment was investigated. A strong positive correlation between *Prochlorococcus* and *Synechococcus* global abundances has been previously interpreted as the result of their physiological similarities and associated similarity in responses to environmental conditions [49]. In our study, size fractionation of the microbes (three size classes; 0.1–20 μm) at selected stations substantially aided in exposing the prominence of *Synechococcus* ecotypes in the Indian Ocean and total dominance of large *Synechococcus* ecotypes in coastal areas and in the largest size fraction (3–20 μm). Additionally some novel and conspicuous picocyanobacterial clades, restricted to the smallest size fraction (0.1–0.8 μm) were identified.

Comparison of picocyanobacterial reads encoding proteins related to energy metabolism underscore differences in phycobiliproteins and nitrate utilization (*nar*B) between *Synechococcus* and *Prochlorococcus*, and significantly expands previous investigations in laboratory strains and strains living in other oceans (*see e*.*g*. [50–51]). Even though recent work on *nar*B-containing *Prochlorococcus* isolates and genomes show that these strains are phylogenetically diverse and may utilize nitrate as a sole nitrogen source [52], our data suggest that this is likely not the case for *Prochlorococccus* strains in Indian Ocean surface waters. Indeed, the capacity to use different nitrogen sources is generally considered as a trait explaining the dominance of *Synechococcus* in coastal waters with higher concentration of nitrate, apparently is also the case in Indian Ocean *Synechoccoccus* ecotypes.

The detection of phycoerthrin genes *cpe*B and *cpe*S in Indian Ocean *Prochlorococcus* phylotypes also stresses the importance of these phycobiliprotein genes (Fig 3B; S1C Fig). These may function as photoreceptors, rather than in light-harvesting, as shown previously in the HL adapted *Prochlorococcus* strain MED4 [17] The *Prochlorococcus cpe*B genes retrieved here are also genetically related to the Red Sea HL *Prochlorococcus* strain AS9601 [53].

A subset of the Indian Ocean stations, in which metagenomic sequencing of picocyanobacteria was conducted on all three size fractions, were also used to obtain deeper insight into key events leading to their contemporary genome size distributions and ecological habitat preferences. From these data it is readily apparent that the Indian Ocean picocyanobacteria have diversified several times. For instance, our analyses suggest that contemporary small-celled *Prochlorococcus* with streamlined genomes (Chl *a* and *b* but absence of phycobilisomes) originated from a cyanobacterial ancestor, sister to contemporary coastal *Synechococcus* ecotypes with large genomes (Chl *a* and light-harvesting phycobilisomes). This evolutionary scenario may have proceeded along two major lines of descent; some *Synechococcus* individuals made their way into open waters while maintaining their larger genomes and cell size. This is verified from our analysis of the *Synechococcus isi*A-like (or CP43´-like) gene, which demonstrates relatedness between contemporary *Synechococcus* and a *Synechococcus* ancestor adapted to iron limitation (*e*.*g*. at GS112). An additional evolutionary lineage proceeded towards the most ecologically successful sister lineage, characterized by small cells and reduced genomes: the genus *Prochlorococcus* as we know today [54]. This radiation into *Prochlorococcus* may have been a monophyletic event (catalyzed by loss of phycobilisome genes) as also suggested by the reconstruction based on the Chl-binding protein genes (Fig 3A). An additional piece of evidence discovered in the habitat reconstruction analyses (Fig 3A–3C, Open ocean vs. Coastal) further suggests that coastal habitats were later re-colonized by small celled strains of extant *Prochlorococcus* retaining their small cell size. Considering the general occurrence of phycobilisomes in *Synechococcus*, it is proposed here that phycobilisomes within putative *Prochlorococcus*-type decedents were either lost or, as in a few representative ecotypes, were retained as minimal phycobilisome-like ‘antenna’ representing a relic of a *cpe*B gene (subunit of phycoerythrin) of the *Synechococcus*-like ancestor. Indeed, other phylogenetic analyses have also suggested a common ancestor (*Synechococcus*-like) of the picocyanobacterial radiation [34, 55, 56].

The phycoerythrin *cpe*B gene relic of contemporary Indian Ocean *Prochlorococcus* is most closely related to *cpe*B genes in HLII adapted *Prochlorococcus* ecotypes found in other oceans [45]. The extent to which the *cpe*B gene of any *Prochlorococcus* is functional is not clear [51]. However, Steglich et al. [20] demonstrated *cpe*B transcription in *Prochlorococcus* MED4 and suggested a function related to transcriptional control or as photoreceptor. Furthermore, as opposed to in *Synechococcus*, Chl *b* was ‘recruited’ in *Prochlorococcus* at some stage, a phenomenon which has occurred several times within cyanobacterial prochlorophytes [57]. As the Indian Ocean *cpe*B containing *Prochlorococcus* ecotypes are phylogenetically related to others with low levels of divinyl Chl *b/*a, such as HL adapted *Prochlorococcus*, it is possible that the *cpe*B gene product either compensates for this loss of light-energy capture or that the *cpe*B gene product has a protective function, such as phycoerythin in *Synechococcus* [40]. This evolutionary scenario among Indian Ocean picocyanobacteria may in turn have been driven and/or facilitated by drastic changes that took place in ocean chemistry some 550 MY ago, characterized by falling iron levels and ocean oxygenation [58]. *Prochlorococcus* and some *Synechococcus* like ecotypes were able to adapt through Fe-saving gene replacements, such as substituting FeSOD with NiSOD [59], and being largely devoid of competitors in the ultra-oligotrophic open Indian Ocean waters, they were able to flourish eventually leading to the phenomenal global distribution and ecological significance of *Prochlorococcus* of today. The fact that the picocyanobacterial phylotypes adapted to the Indian Ocean (represented here as GS112; [17]) have numerous counterparts in other coastal and open waters stations from both at the Atlantic and Pacific basins examined during GOSI suggests that a similar picocyanobacterial evolutionary scenario may have bearing in a general global ocean context.

## Supporting Information

S1 Fig The distribution of picocyanobacteria in the Indian Ocean in relation to priority stations and size fractions. Closest BLAST hits are given to the right of the heat maps. A. Chlorophyll-binding proteins (Pcb/IsiA) and B, Phycobilisome α-subunits and C, phycobilisome ß-subunits of *Prochlorococcus* and *Synechococcus*.
(EPS)

S2 Fig Pcb/IsiA, light-harvesting chlorophyll-binding peptides rooted trees. The trees were inferred using maximum likelihood showing samples from Indian Ocean, Atlantic and Pacific locations (colored dots), taxonomy (red: *Synechococcus*; blue: *Prochlorococcus*), and the closer match to the database from each sequenced. Scale bar indicates expected number of substitutions per site.
(EPS)

S3 Fig Light-harvesting phycobilisome β-subunits (*cpe*, *cpc*, *apc*) rooted trees. The trees were inferred using maximum likelihood showing samples from Indian Ocean, Atlantic and Pacific locations (colored dots), taxonomy (red: *Synechococcus*; blue: *Prochlorococcus*), and the closer match to the database from each sequenced. Scale bar indicates expected number of substitutions per site.
(EPS)

S4 Fig Light-harvesting phycobilisome α-subunits (*cpe*, *cpc*, *apc*) rooted trees. The trees were inferred using maximum likelihood showing samples from Indian Ocean, Atlantic and Pacific locations (colored dots), taxonomy (red: *Synechococcus*; blue: *Prochlorococcus*), and the closer match to the database from each sequenced. Scale bar indicates expected number of substitutions per site.
(EPS)

S5 Fig The distribution of ‘Energy metabolism’ related KEGG Orthology (KO) categories in the *Prochlorococcus* and *Synechococcus* picocyanobacteria at the Indian Ocean. KEGG categories and their relative abundances are given per size fraction at the Indian Ocean stations GS108, GS110, GS112, GS117 and GS122.
(EPS)

S6 Fig Distribution of genes related to nutrient and oxidative stress in cyanobacteria. The sensor-response histidine kinase encoding genes, involved in signaling phycobilisomes degradation under low nutrient conditions (*nbl*S, K07769) and high light stress (*hli*A (putative high light inducible protein)), as well as genes for the putative nickel-containing superoxide dismutase (*sod*N, K00518) and nitrate reductase (*nar*B, K00367) genes in the different size fractions. Number of reads were normalized to *recA*.
(EPS)

S1 Table Metadata for the 18 sampling stations along the east-west Indian Ocean transect, and the Atlantic and Pacific stations including for comparisons at the present study. (DOCX)

S2 Table Light harvesting gene sequences included as references in Fig 3, numbered from 1 and downwards. (DOCX)

S3 Table HOMOVA test [36] as implemented in MOTHUR, v.1.33.3 [37], based on the JTT-sequence distances calculated in PROTDIST [38]. (DOCX)

S4 Table AMOVA test [36] as implemented in MOTHUR, v.1.33.3 [37], based on the JTT-sequence distances calculated in PROTDIST [38]. (DOCX)
