# Supplementary material for: Metagenomic Analysis of the Indian Ocean Picocyanobacterial Community: Structure, Potential Function and Evolution
Source: PLoS One. 2016 May 19;11(5):e0155757. doi: 10.1371/journal.pone.0155757 (PMC4890579; doi:10.1371/journal.pone.0155757)
Supplement: S1 Table — (DOCX) [file pone.0155757.s007.docx]

| **Station** | **Region** | **Date**  **(d/m/y)** | **Coordenates** | **Depth**  **(m)** | **Sample Depth (m)** | **T (ºC)** | **Sal**  **(psu)** | **O_2_** | **pH** | **Turbidity** | **Habitat** | **Chl *a***  **(mg/L)** | **TDN^a, b^** | **NO_3_+ NO_2_^b^** | **NH_4_^b^** | **DON^b^** | **Urea^b^** | **PO_4_^c^** | **Si^d^** | **DIN:PO_4_** | **Source** |
| --- | --- | --- | --- | --- | --- | --- | --- | --- | --- | --- | --- | --- | --- | --- | --- | --- | --- | --- | --- | --- | --- |
| GS108 | Indian Ocean | 04/08/05 | 12°5'33"S, 96°52'54"E | 7 | 1,8 | 25,9 | 32,4 | 4,8 | 8,0 | 7,2 | Reef | 0,108 | NA | 0,660 | 0,000 | NA | 0,535 | 1,235 | 1,381 | 0,53 | this study |
| GS109 | Indian Ocean | 05/08/05 | 10°56'37"S, 92°3'32"E | 4573 | 1,5 | 27,2 | 32,6 | 4,16 | 7,8 | 7 | Open Ocean | 0,138 | NA | 0,246 | 0,000 | NA | 2,111 | 0,193 | 1,401 | 1,27 | this study |
| GS110 | Indian Ocean | 06/08/05 | 10°26'46"S, 88°18'10"E | 1219 | 1,5 | 27 | 32,7 | 4,13 | 7,7 | 6,8 | Open Ocean | 0,128 | 3,75 | 0,258 | 0,067 | 3,433 | 0,846 | 0,162 | 1,341 | 2,01 | this study |
| GS111 | Indian Ocean | 07/08/05 | 9°35'49"S, 84°11'51"E | 3841 | 1,8 | 26,4 | 32,3 | 4,22 | 7,8 | 7 | Open Ocean | 0,203 | 4,33 | 0,388 | 0,164 | 3,785 | 2,264 | 0,349 | 1,144 | 1,58 | this study |
| GS112 | Indian Ocean | 08/08/05 | 8°30'18"S, 80°22'32"E | 4573 | 1,8 | 26,6 | 32,5 | 4,85 | 7,8 | 7 | Open Ocean | 0,134 | 4,35 | 0,374 | 0,283 | 3,702 | 0,950 | 0,610 | 1,644 | 1,08 | this study |
| GS113 | Indian Ocean | 09/08/05 | 7°0'27"S,  76°19'53"E | 4573 | 1,8 | 27,5 | 33,3 | 4,51 | 7,8 | 7 | Open Ocean | 0,24 | 4,11 | 0,723 | 0,000 | 3,394 | 2,255 | 0,386 | 0,963 | 1,87 | this study |
| GS114 | Indian Ocean | 15/08/05 | 4°59'25"S, 64°58'36"E | 3649 | 1,5 | 28,2 | 33,1 | 4,54 | 7,9 | 7 | Open Ocean | 0,14 | 2,39 | 0,184 | 0,367 | 1,839 | 0,770 | 0,452 | 0,969 | 1,22 | this study |
| GS115 | Indian Ocean | 16/08/05 | 4°39'45"S, 60°31'23"E | 3219 | 1,5 | 27,9 | 33,2 | 4,93 | 8,0 | 6,9 | Open Ocean | 0,142 | 3,70 | 0,405 | 0,000 | 3,303 | 0,314 | 0,540 | 1,198 | 0,75 | this study |
| GS116 | Indian Ocean | 17/08/05 | 4°38'6"S,  56°50'10"E | 2149 | 1,5 | 26,2 | 33,1 | 5,01 | 8 | 6,8 | Open Ocean | 0,287 | 4,78 | 0,429 | 0,025 | 4,332 | 3,088 | 0,457 | 1,835 | 0,99 | this study |
| GS117 | Indian Ocean | 09/09/05 | 4°36'49"S, 55°30'31"E | 14 | 1,8 | 26,4 | 35,5 | 6,01 | 8,2 | 7,2 | Coastal | 0,206 | 4,61 | 0,397 | 0,000 | 4,213 | 3,267 | 0,133 | 1,289 | 2,98 | this study |
| GS118 | Indian Ocean | 10/09/05 | 10°37'12"S, 56°54'46"E | 4206 | 1,8 | 25,5 | 35,2 | 5,92 | 8,2 | 6,7 | Open Ocean | NA | 4,21 | 0,799 | 0,303 | 3,115 | 1,512 | 0,412 | 1,847 | 2,67 | this study |
| GS119 | Indian Ocean | 26/09/05 | 23°12'58"S, 52°18'22"E | 2994 | 1,9 | 23,8 | 35,4 | 4,29 | 8,1 | 6,5 | Open Ocean | 0,078 | 2,97 | 0,231 | 0,000 | 2,742 | 0,636 | 0,107 | 1,184 | 2,16 | this study |
| GS120 | Indian Ocean | 27/09/05 | 26°2'6"S,  50°7'23"E | 5081 | 2,8 | 22,5 | 35,6 | 5,79 | 8,1 | 6,5 | Open Ocean | 0,117 | 5,70 | 0,138 | 0,047 | 5,523 | 0,307 | 1,030 | 0,706 | 0,18 | this study |
| GS121 | Indian Ocean | 29/09/05 | 29°20'56"S, 43°12'56"E | 4309 | 1,5 | 23,1 | 35,4 | 5,59 | 8,2 | 6,7 | Open Ocean | 0,14 | 2,80 | 0,188 | 0,000 | 2,620 | 0,595 | 0,387 | 0,752 | 0,49 | this study |
| GS122 | Indian Ocean | 30/09/05 | 30°53'54"S, 40°25'13"E | 4921 | 1,9 | 20,2 | 35,8 | 6,57 | 8,2 | 6,4 | Open Ocean | 0,151 | 3,08 | 0,146 | 0,000 | 2,941 | 0,541 | 0,182 | 1,258 | 0,80 | this study |
| GS123 | Indian Ocean | 01/10/05 | 32°23'57"S, 36°35'31"E | 1860 | 2,2 | 20,4 | 35,8 | 6,16 | 8,2 | 6,5 | Open Ocean | 0,228 | 2,71 | 0,348 | 0,000 | 2,364 | 3,446 | 0,769 | 0,832 | 0,45 | this study |
| GS148 | Indian Ocean | 11/09/05 | 6°19'S,  39°33'E | 1 | 0,3 | 25,7 | 35,2 | NA | NA | NA | Reef | NA | 4,19 | NA | NA | NA | 0,150 | 0,074 | 1,787 | NA | this study |
| GS149 | Indian Ocean | 12/09/05 | 6° 7'S, 3  9°07'E | 5 | 1,5 | 25,7 | 35,2 | NA | NA | NA | Harbor | NA | 4,35 | NA | NA | NA | 0,181 | 0,065 | 1,638 | NA | this study |
| GS000 | Sargasso Sea | 26/02/03 | 31º10'30"N, 64º19'27.6"W | >4200 | 5 | 20,5 | 36,7 | 5,1 | NA | NA | Open Ocean | 0,17 | NA | 0.089 | NA | NA | NA | 0.058 | 0.84 | NA | Rusch et al. 2007 |
| GS001 | Sargasso Sea | 15/05/03 | 32º10'00"N, 64º30'00"W | >4200 | 5 | 22,9 | 36,7 | 5,1 | NA | NA | Open Ocean | 0,10 | NA | 0,063 | NA | NA | NA | 0,1 | 0,9 | NA | Rusch et al. 2007 |
| GS002 | North American East Coast | 21/08/03 | 42º30'11"N, 67º14'24"W | 106 | 1 | 18,2 | 29,2 | 13,9 | NA | NA | Coastal | 1,4 | NA | 0,05 | NA | NA | NA | 1,4 | 3,2 | NA | Rusch et al. 2007 |
| GS003 | North American East Coast | 21/08/03 | 42º51'10"N, 66º13'2"W | 119 | 1 | 11,7 | 29,9 | 16,2 | NA | NA | Coastal | 1,4 | NA | 0,13 | NA | NA | NA | 1,4 | 6,1 | NA | Rusch et al. 2007 |
| GS004 | North American East Coast | 22/08/03 | 44º8'14"N, 63º38'40"W | 142 | 2 | 17,3 | 28,3 | 13,6 | NA | NA | Coastal | 0,4 | NA | 0,05 | NA | NA | NA | 0,4 | 1,2 | NA | Rusch et al. 2007 |
| GS005 | North American East Coast | 22/08/03 | 44º41'25"N, 63º38'14"W | 64 | 1 | 15 | 30,2 | 5,8 | NA | NA | Embayment | 6 | NA | 1,1 | NA | NA | NA | 6 | 1,8 | NA | Rusch et al. 2007 |
| GS006 | North American East Coast | 23/08/03 | 45º6'42"N, 64º56'48"W | 11 | 1 | 11,2 | 31,1 | 5,8 | NA | NA | Estuary | 2,8 | NA | 5,3 | NA | NA | NA | 2,8 | 7,6 | NA | Rusch et al. 2007 |
| GS007 | North American East Coast | 25/08/03 | 43º37'56"N, 66º50'50"W | 139 | 1 | 17,9 | 31,7 | 6,4 | NA | NA | Coastal | 1,4 | NA | 0,14 | NA | NA | NA | 1,4 | 5,9 | NA | Rusch et al. 2007 |
| GS008 | North American East Coast | 16/11/03 | 41º29'9"N, 71º21'4"W | 12 | 1 | 9,4 | 26,5 | 3,4 | NA | NA | Coastal | 2,2 | NA | 4,9 | NA | NA | NA | 2,2 | 2,2 | NA | Rusch et al. 2007 |
| GS009 | North American East Coast | 17/11/03 | 41º5'28"N, 71º36'8"W | 32 | 1 | 11 | 31 | 3,4 | NA | NA | Coastal | 4,0 | NA | 4,9 | NA | NA | NA | 4 | 2,2 | NA | Rusch et al. 2007 |
| GS010 | North American East Coast | 18/11/03 | 38º56'24"N, 74º41'6"W | 10 | 1 | 12 | 31 | 3,5 | NA | NA | Coastal | 2,0 | NA | 2 | NA | NA | NA | 2 | 0,59 | NA | Rusch et al. 2007 |
| GS011 | North American East Coast | 18/11/03 | 39º25'4"N, 75º30'15"W | 8 | 1 | 11 | 34,8 | 4,5 | NA | NA | Estuary | 4,8 | NA | 2,2 | NA | NA | NA | 4,8 | 0,68 | NA | Rusch et al. 2007 |
| GS012 | North American East Coast | 18/12/03 | 38º56'49"N, 76º25'2"W | 25 | 13,2 | 1 | 3,5 | 5,7 | NA | NA | Estuary | 21,0 | NA | 3,5 | NA | NA | NA | 21 | 0,82 | NA | Rusch et al. 2007 |
| GS013 | North American East Coast | 19/12/03 | 36º0'14"N, 75º23'41"W | 20 | 2,1 | 9,3 | 35,2 | 4,4 | NA | NA | Coastal | 3,0 | NA | 4,5 | NA | NA | NA | 3 | 2,4 | NA | Rusch et al. 2007 |
| GS014 | North American East Coast | 20/12/03 | 32º30'25"N, 79º15'50"W | 31 | 1 | 18,6 | 36,3 | 3,8 | NA | NA | Coastal | 1,70 | NA | 0,28 | NA | NA | NA | 1,7 | 2,1 | NA | Rusch et al. 2007 |
| GS015 | Caribbean Sea | 08/01/04 | 24º29'18"N, 83º4'12"W | 47 | 1,7 | 25 | 36 | 5,6 | NA | NA | Coastal | 0,2 | NA | 1,1 | NA | NA | NA | 0,2 | 1,3 | NA | Rusch et al. 2007 |
| GS016 | Caribbean Sea | 08/01/04 | 24º10'29"N, 84º20'40"W | 3333 | 2 | 26,4 | 35,8 | 5,8 | NA | NA | Coastal | 0,16 | NA | 1,1 | NA | NA | NA | 0,16 | 1,3 | NA | Rusch et al. 2007 |
| GS017 | Caribbean Sea | 09/01/04 | 20º31'21"N, 85º24'49"W | 4513 | 2 | 27 | 35,8 | 6 | NA | NA | Open Ocean | 0,13 | NA | 0,2 | NA | NA | NA | 0,13 | 2 | NA | Rusch et al. 2007 |
| GS018 | Caribbean Sea | 10/01/04 | 18º2'12"N, 83º47'5"W | 4470 | 1,7 | 27,4 | 35,4 | 5,6 | NA | NA | Open Ocean | 0,14 | NA | 0,59 | NA | NA | NA | 0,14 | 2,2 | NA | Rusch et al. 2007 |
| GS019 | Caribbean Sea | 12/01/04 | 10º42'59"N, 80º15'16"W | 3336 | 1,7 | 27,7 | 35,4 | 6,4 | NA | NA | Coastal | 0,23 | NA | 0 | NA | NA | NA | 0,23 | 2,2 | NA | Rusch et al. 2007 |
| GS020 | Panama Canal | 15/01/04 | 9º9'52"N, 79º50'10"W | 4.2 | 2 | 28,6 | 0,1 | 5,8 | NA | NA | Fresh Water | NA | NA | 4,2 | NA | NA | NA | N/A | 169,5 | NA | Rusch et al. 2007 |
| GS021 | Eastern Tropical Pacific | 19/01/04 | 8º7'45"N, 79º41'28"W | 76 | 1,6 | 27,6 | 30,7 | 6,4 | NA | NA | Coastal | 0,50 | NA | 0,36 | NA | NA | NA | 0,5 | 1,1 | NA | Rusch et al. 2007 |
| GS022 | Eastern Tropical Pacific | 20/01/04 | 6º29'34"N, 82º54'14"W | 2431 | 2 | 29,3 | 32,3 | 5,4 | NA | NA | Open Ocean | 0,33 | NA | 0,17 | NA | NA | NA | 0,33 | 1,3 | NA | Rusch et al. 2007 |
| GS023 | Eastern Tropical Pacific | 21/01/04 | 5º38'24"N, 86º33'55"W | 1139 | 2 | 28,7 | 32,6 | 6,5 | NA | NA | Open Ocean | 0,07 | NA | 0,052 | NA | NA | NA | 0,07 | 1,1 | NA | Rusch et al. 2007 |
| GS025 | Eastern Tropical Pacific | 20/01/04 | 5º33'10"N, 87º5'16"W | 30 | 1,1 | 28,3 | 31,4 | 6,5 | NA | NA | Fringing Reef | 0,11 | NA | 0,11 | NA | NA | NA | 0,11 | 0,73 | NA | Rusch et al. 2007 |
| GS026 | Galapagos Islands | 01/02/04 | 1º15'51"N, 90º17'42"W | 2386 | 2 | 27,8 | 32,6 | 6 | NA | NA | Open Ocean | 0,22 | NA | 0,33 | NA | NA | NA | 0,22 | 0,33 | NA | Rusch et al. 2007 |
| GS027 | Galapagos Islands | 04/02/04 | 1º12'58"S, 90º25'22"W | 2.3 | 2,2 | 25,5 | 34,9 | 6,4 | NA | NA | Coastal | 0,40 | NA | 5,5 | NA | NA | NA | 0,4 | 1,2 | NA | Rusch et al. 2007 |
| GS028 | Galapagos Islands | 04/02/04 | 1º13'1"S, 90º19'11"W | 156 | 2 | 25,6 | 34 | 4,5 | NA | NA | Coastal | 0,35 | NA | 5,8 | NA | NA | NA | 0,35 | 1 | NA | Rusch et al. 2007 |
| GS029 | Galapagos Islands | 08/02/04 | 0º12'0"S, 90º50'7"W | 12 | 2,1 | 26,2 | 34,5 | 5,2 | NA | NA | Coastal | 0,40 | NA | 3,3 | NA | NA | NA | 0,4 | 1,7 | NA | Rusch et al. 2007 |
| GS030 | Galapagos Islands | 09/02/04 | 0º16'20"N, 91º38'0"W | 19 | 19 | 26,9 | 34,5 | 4,5 | NA | NA | Warm Seep | 0,47 | NA | 0,71 | NA | NA | NA | 0,47 | 1,2 | NA | this study |
| GS031 | Galapagos Islands | 10/02/04 | 0º18'4"S, 91º39'6"W | 19.6 | 12 | 18,6 | 34,9 | 3,3 | NA | NA | Coastal upwelling | 0,35 | NA | 11,7 | NA | NA | NA | 0,35 | 4,7 | NA | Rusch et al. 2007 |
| GS032 | Galapagos Islands | 11/02/04 | 0º35'38"S, 91º4'10"W | 1.6 | 0,1 | 25,4 | 33,8 | 2,5 | NA | NA | Mangrove | 3,8 | NA | 11,3 | NA | NA | NA | 3,8 | 76,8 | NA | this study |
| GS033 | Galapagos Islands | 19/02/04 | 1º13'42"S, 90º25'45"W | 0.3 | 0,2 | 37,6 | 33,8 | 0,6 | NA | NA | Hypersaline | 0,31 | NA | 0,23 | NA | NA | NA | 0,31 | 45,4 | NA | this study |
| GS034 | Galapagos Islands | 19/02/04 | 0º22'59"S, 90º16'47"W | 35 | 2,1 | 27,5 | 33,8 | 5,5 | NA | NA | Coastal | 0,36 | NA | 2,2 | NA | NA | NA | 0,36 | 1,2 | NA | Rusch et al. 2007 |
| GS035 | Galapagos Islands | 01/03/04 | 1º23'21"N, 91º49'1"W | 71 | 1,7 | 21,8 | 34,5 | 7,4 | NA | NA | Coastal | 0,28 | NA | 7,4 | NA | NA | NA | 0,28 | 2,6 | NA | Rusch et al. 2007 |
| GS036 | Galapagos Islands | 02/03/04 | 0º1'15"S, 91º11'52"W | 67 | 2,1 | 25,8 | 34,6 | 8,5 | NA | NA | Coastal | 0,65 | NA | 2,9 | NA | NA | NA | 0,65 | 1,9 | NA | Rusch et al. 2007 |
| GS037 | Eastern Tropical Pacific | 17/03/04 | 1º58'26"S, 95º0'53"W | 3334 | 1,8 | 28 | 34,2 | 7,9 | NA | NA | Open Ocean | 0,21 | NA | 4,1 | NA | NA | NA | 0,21 | 1,7 | NA | Rusch et al. 2007 |
| GS038 | Tropical South Pacific | 18/03/04 | 2º34'55"S, 97º51'5"W | >4000 | 1,8 | 28,4 | 34,2 | 8 | NA | NA | Open Ocean | 0,23 | NA | 3,8 | NA | NA | NA | 0,23 | 1,7 | NA | this study |
| GS039 | Tropical South Pacific | 19/03/04 | 3º20'36"S, 101º22'26"W | >4000 | 2 | 28,6 | 34,6 | 9,1 | NA | NA | Open Ocean | 0,23 | NA | 3,6 | NA | NA | NA | 0,23 | 1,3 | NA | this study |
| GS040 | Tropical South Pacific | 20/03/04 | 4º29'56"S, 105º04'12"W | >4000 | 2,2 | 27,8 | 31,8 | 6,1 | NA | NA | Open Ocean | 0,19 | NA | 2,2 | NA | NA | NA | 0,19 | 1,1 | NA | this study |
| GS041 | Tropical South Pacific | 21/03/04 | 5º55'48"S, 108º41'13"W | >4000 | 2 | 28 | 35 | 6,6 | NA | NA | Open Ocean | 0,17 | NA | 5 | NA | NA | NA | 0,17 | 2 | NA | this study |
| GS042 | Tropical South Pacific | 23/03/04 | 7º06'27"S, 116º07'9"W | >4000 | 1,7 | 27,6 | 38,1 | 5,5 | NA | NA | Open Ocean | 0,14 | NA | 3,2 | NA | NA | NA | 0,14 | 0,7 | NA | this study |
| GS043 | Tropical South Pacific | 24/03/04 | 7º39'40"S, 120º24'8"W | >4000 | 1,9 | 27,6 | 35,9 | 6,7 | NA | NA | Open Ocean | 0,14 | NA | 4,8 | NA | NA | NA | 0,14 | 1,7 | NA | this study |
| GS044 | Tropical South Pacific | 25/03/04 | 8º24'54"S, 124º14'23"W | >4000 | 2 | 27,6 | 39,4 | 7 | NA | NA | Open Ocean | 0,12 | NA | 5 | NA | NA | NA | 0,12 | 1,9 | NA | this study |
| GS045 | Tropical South Pacific | 26/03/04 | 9º01'3"S, 127º46'2"W | >4000 | 1,7 | 28,3 | 37 | 7 | NA | NA | Open Ocean | 0,13 | NA | 3,8 | NA | NA | NA | 0,13 | 1,4 | NA | this study |
| GS046 | Tropical South Pacific | 27/03/04 | 9º34'16"S, 131º29'30"W | >4000 | 1,9 | 28,7 | 35,6 | 6,8 | NA | NA | Open Ocean | 0,13 | NA | 3,2 | NA | NA | NA | 0,13 | 1,1 | NA | this study |
| GS047 | Tropical South Pacific | 28/03/04 | 10º7'53"S, 135º26'58"W | 2400 | 30 | 28,6 | 37,3 | 5,6 | NA | NA | Open Ocean | 0,12 | NA | 1,7 | NA | NA | NA | 0,12 | 0,64 | NA | this study |
| GS048 | Tropical South Pacific | 17/05/04 | 17°28'33"S, 149°48'43"W | 2 | 1,25 | 28,9 | 35,1 | 5,7 | 8,2 | NA | Reef | 0,10 | 8,63 | 0,015 | 0,107 | 7,584 | 0,887 | 0,10 | 1,2 | 5,13 | this study |
| GS049 | Tripocal South Pacific | 17/05/04 | 17°27'10"S, 149°47'56"W | 1000 | 1,22 | 28,8 | 32,6 | 4,5 | 8,2 | NA | Coastal | 0,10 | NA | 0,015 | 0,071 | NA | 2,067 | 0,10 | 2,6 | 13,19 | this study |
| GS050 | Polynesia Archipelagos | 19/05/04 | 15º16'40"S, 148º13'28"W | 24 | 1,2 | 27,8 | 36,2 | 4,3 | NA | NA | Coral Atoll | 0,07 | NA | 0,44 | NA | NA | NA | 0,07 | 0,52 | NA | this study |
| GS051 | Polynesia Archipelagos | 22/05/04 | 15º8'37"S, 147º26'6"W | 10 | 1 | 27,3 | 34,2 | 4,8 | NA | NA | Coral Atoll | 0,14 | NA | 0,62 | NA | NA | NA | 0,17 | 0,84 | NA | this study |

**^a^**Total Dissolved Nitrogen

^b^µmol N/L

^c^µmol P/L

^d^µmol Si/L
