# Supplementary material for: Metagenomic Analysis of the Indian Ocean Picocyanobacterial Community: Structure, Potential Function and Evolution
Source: PLoS One. 2016 May 19;11(5):e0155757. doi: 10.1371/journal.pone.0155757 (PMC4890579; doi:10.1371/journal.pone.0155757)
Supplement: S2 Table — (DOCX) [file pone.0155757.s008.docx]

| **Light-harvesting chlorophyll-binding peptides (Pcb/IsiA)** | | | | |
| --- | --- | --- | --- | --- |
| **1** | YP381894.1 | IsiA | Synechococcus sp. CC9605 | |
| **2** | ZP05789774.1 | IsiA | Synechococcus sp. WH 8109 | |
| **3** | ABB92216.1 | IsiA | uncultured marine type-A Synechococcus 5B2 | |
| **4** | YP731503.1 | IsiA | Synechococcus sp. CC9311 | |
| **5** | YP377013.1 | IsiA | Synechococcus sp. CC9902 | |
| **6** | ZP01468016.1 | IsiA | Synechococcus sp. BL107 | |
| **7** | YP001014513.1 | PcbA | Prochlorococcus marinus str. NATL1A | |
| **8** | YP291261.1 | PcbA | Prochlorococcus marinus str. NATL2A | |
| **9** | YP291916.1 | PcbE | Prochlorococcus marinus str. NATL2A | |
| **10** | YP291917.1 | PcbB | Prochlorococcus marinus str. NATL2A | |
| **11** | YP001011009.1 | PcbA | Prochlorococcus marinus str. MIT 9515 | |
| **12** | NP892745.1 | PcbA | Prochlorococcus marinus subsp. pastoris str. CCMP1986 | |
| **13** | YP001009076.1 | PcbA | Prochlorococcus marinus str. subsp. pastoris str. AS9601 | |
| **14** | AF354647.1 | PcbA | Prochlorococcus sp. TAK9803 | |
| **15** | AF354645.1 | Pcb | Prochlorococcus marinus str. SB | |
| **16** | ZP05138915.1 | PcbA | Prochlorococcus marinus str. MIT 9202 | |
| **17** | CAL01054.1 | PcbA | uncultured Prochlorococcus sp. | |
| **18** | CAL01039.1 | PcbA | uncultured Prochlorococcus sp. | |
| **19** | CAL01097.1 | PcbC | uncultured Prochlorococcus sp. | |
| **20** | YP001014670.1 | PcbD | Prochlorococcus marinus str. NATL1A | |
| **21** | YP397766.1 | PcbD | Prochlorococcus marinus str. MIT 9312 | |
| **22** | YP001011656.1 | PcbD | Prochlorococcus marinus str. MIT 9515 | |
| **23** | YP001009754.1 | PcbD | Prochlorococcus marinus str. AS9601 | |
| **24** | YP001091596.1 | PcbD | Prochlorococcus marinus str. MIT 9301 | |
| **25** | YP001484585.1 | PcbD | Prochlorococcus marinus str. MIT 9215 | |
| **26** | ABE10784.1 | PcbD | Prochlorococcus marinus clone ASNC1363 | |
| **Light harvesing phycobilidome beta-subunits (cpe, cpc, apc)** | | | | |
| **1** | YP381516 | ApcB | Synechococcus sp. CC9605 | |
| **2** | NP897167 | ApcB | Synechococcus sp. WH8102 | |
| **3** | YP001225069 | ApcB | Synechococcus sp. WH 7803 | |
| **4** | YP382492 | ApcB | Synechococcus sp. CC9605 | |
| **5** | NP896579 | ApcB | Synechococcus sp. WH8102 | |
| **6** | ZP01081253 | ApcB | Synechococcus sp. RS9917 | |
| **7** | ZP01473073 | CpcB | Synechococcus sp. RS9916 | |
| **8** | NP898113 | CpcB | Synechococcus sp. WH 8102 | |
| **9** | YP380752 | CpcB | Synechococcus sp. CC9605 | |
| **10** | YP377894 | CpeB | Synechococcus sp. CC9902 | |
| **11** | YP380766 | CpeB | Synechococcus sp. CC9605 | |
| **12** | NP898099 | CpeB | Synechococcus sp. WH 8102 | |
| **13** | CAB75589 | CpeB | Prochlorococcus marinus str. PAC1 | |
| **14** | YP377904 | CpeB | Synechococcus sp. CC9902 | |
| **15** | ZP01469884 | CpeB | Synechococcus sp. BL107 | |
| **16** | YP380757 | CpeB | Synechococcus sp. CC9605 | |
| **17** | NP898108 | CpeB | Synechococcus sp. WH 8102 | |
| **18** | YP001010655 | CpeB | Prochlorococcus marinus str. MIT 9515 | |
| **19** | NP892424 | CpeB | Prochlorococcus marinus subsp. pastoris str. CCMP1986 | |
| **20** | ABE10844 | CpeB | Prochlorococcus marinus clone ASNC2150 | |
| **21** | YP001090554 | CpeB | Prochlorococcus marinus str. MIT 9301 | |
| **22** | CAC83121 | CpeB | Prochlorococcus marinus str. TAK9803-2 | |
| **23** | YP396804 | CpeB | Prochlorococcus marinus str. MIT 9312 | |
| **24** | YP001008724 | CpeB | Prochlorococcus marinus str. AS9601 | |
| **25** | ZP05138202 | CpeB | Prochlorococcus marinus str. MIT 9202 | |
| **Light harvesing phycobilidome alpha-subunits (cpe, cpc, apc)** | | | | |
| **1** | YP001228313 | CpeA class I | | Synechococcus sp. RCC307 |
| **2** | Q02179 | CpeA class I | | Synechococcus sp. WH 8020 |
| **3** | CBX53435 | CpeA | | uncultured cyanobacterium |
| **4** | CBX53455 | CpeA | | uncultured cyanobacterium |
| **5** | NP898107 | CpeA class I | | Synechococcus sp. WH 8102 |
| **6** | YP380758 | CpeA class I | | Synechococcus sp. CC9605 |
| **7** | ZP01473089 | CpeA class II | | Synechococcus sp. RS9916 |
| **8** | YP377895 | CpeA class II | | Synechococcus sp. CC9902 |
| **9** | NP898100 | CpeA class II | | Synechococcus sp. WH 8102 |
| **10** | AFK25754 | CpeA class II | | Synechococcus sp. M11.2 |
| **11** | YP380765 | CpeA class II | | Synechococcus sp. CC9605 |
| **12** | NP896580 | ApcA | | Synechococcus sp. WH 8102 |
| **13** | YP382491 | ApcA | | Synechococcus sp. CC9605 |
| **14** | ZP05788646 | ApcA | | Synechococcus sp. WH 8109 |
| **15** | ZP01122883 | ApcA | | Synechococcus sp. WH 7805 |
| **16** | YP380751 | CpcA | | Synechococcus sp. CC9605 |
| **17** | ZP05788292 | CpcA | | Synechococcus sp. WH 8109 |
| **18** | P11394 | CpcA | | Synechococcus sp. WH 8103 |
| **19** | ZP08957395 | CpcA | | Synechococcus sp. WH 8016 |
| **20** | YP377910 | CpcA | | Synechococcus sp. CC9902 |
