# Supplementary material for: Metagenomic Analysis of the Indian Ocean Picocyanobacterial Community: Structure, Potential Function and Evolution
Source: PLoS One. 2016 May 19;11(5):e0155757. doi: 10.1371/journal.pone.0155757 (PMC4890579; doi:10.1371/journal.pone.0155757)
Supplement: S3 Table — (DOCX) [file pone.0155757.s009.docx]

| **HOMOVA test**  **Light harvesting phycobilisome alpha-subunit** | **BValue** | **P-value** | **SSwithin/(Ni-1)_values** | | |  |  |  |  |
| --- | --- | --- | --- | --- | --- | --- | --- | --- | --- |
| Eastern_Tropical_Pacific-Galapagos-Indian_Ocean-Polynesia_Archipelagos-Sargasso-Tropical_South_Pacific | 67.404 | 0.075 | 0.353021 | 0.800653 | 4.62549 | 0.800209 | 0.741746 | 0.782594 |  |
| **HOMOVA test**  **Light harvesting phycobilisome beta-subunit** | **BValue** | **P-value** | **SSwithin/(Ni-1)_values** | | |  |  |  |  |
| Atlantic-Eastern_Tropical_Pacific-Galapagos-Indian_Ocean-Polynesia_Archipelagos-Sargasso-Tropical_South_Pacific | 48.7801 | 0.253 | 0.515992 | 0.339192 | 0.809361 | 0.546803 | 0.939858 | 1.4612 | 1.39669 |
| **HOMOVA test**  **Chlorophyll-binding peptides (Pcb/IsiA)** | **BValue** | **P-value** | **SSwithin/(Ni-1)_values** | | |  |  |  |  |
| Atlantic-Eastern_Tropical_Pacific-Galapagos-Indian_Ocean-Polynesia_Archipelagos-Sargasso-Tropical_South_Pacific | 207.59 | 0.002* | 8.21962 | 1.80586 | 0.263278 | 1.76525 | 1.18993 | 2.0345 | 1.93993 |
| Atlantic-Eastern_Tropical_Pacific | 34.8249 | 0.016 | 8.21962 | 1.80586 |  |  |  |  |  |
| Atlantic-Galapagos | 56.5946 | <0.001* | 8.21962 | 0.263278 |  |  |  |  |  |
| Atlantic-Indian_Ocean | 134.988 | <0.001* | 8.21962 | 1.76525 |  |  |  |  |  |
| Atlantic-Polynesia_Archipelagos | 44.0256 | 0.005 | 8.21962 | 1.18993 |  |  |  |  |  |
| Atlantic-Sargasso | 29.7445 | 0.009 | 8.21962 | 2.0345 |  |  |  |  |  |
| Atlantic-Tropical_South_Pacific | 61.6456 | <0.001* | 8.21962 | 1.93993 |  |  |  |  |  |
| Eastern_Tropical_Pacific-Galapagos | 22.1809 | 0.167 | 1.80586 | 0.263278 |  |  |  |  |  |
| Eastern_Tropical_Pacific-Indian_Ocean | 0.0126013 | 0.987 | 1.80586 | 1.76525 |  |  |  |  |  |
| Eastern_Tropical_Pacific-Polynesia_Archipelagos | 2.13134 | 0.675 | 1.80586 | 1.18993 |  |  |  |  |  |
| Eastern_Tropical_Pacific-Sargasso | 0.195367 | 0.875 | 1.80586 | 2.0345 |  |  |  |  |  |
| Eastern_Tropical_Pacific-Tropical_South_Pacific | 0.0982966 | 0.921 | 1.80586 | 1.93993 |  |  |  |  |  |
| Galapagos-Indian_Ocean | 24.3696 | 0.427 | 0.263278 | 1.76525 |  |  |  |  |  |
| Galapagos-Polynesia_Archipelagos | 14.2271 | 0.293 | 0.263278 | 1.18993 |  |  |  |  |  |
| Galapagos-Sargasso | 24.4234 | 0.235 | 0.263278 | 2.0345 |  |  |  |  |  |
| Galapagos-Tropical_South_Pacific | 25.2696 | 0.341 | 0.263278 | 1.93993 |  |  |  |  |  |
| Indian_Ocean-Polynesia_Archipelagos | 2.85718 | 0.701 | 1.76525 | 1.18993 |  |  |  |  |  |
| Indian_Ocean-Sargasso | 0.497845 | 0.853 | 1.76525 | 2.0345 |  |  |  |  |  |
| Indian_Ocean-Tropical_South_Pacific | 0.430716 | 0.805 | 1.76525 | 1.93993 |  |  |  |  |  |
| Polynesia_Archipelagos-Sargasso | 3.47112 | 0.593 | 1.18993 | 2.0345 |  |  |  |  |  |
| Polynesia_Archipelagos-Tropical_South_Pacific | 3.69345 | 0.617 | 1.18993 | 1.93993 |  |  |  |  |  |
| Sargasso-Tropical_South_Pacific | 0.0435509 | 0.946 | 2.0345 | 1.93993 |  |  |  |  |  |
