# Supplementary material for: Metagenomic Analysis of the Indian Ocean Picocyanobacterial Community: Structure, Potential Function and Evolution
Source: PLoS One. 2016 May 19;11(5):e0155757. doi: 10.1371/journal.pone.0155757 (PMC4890579; doi:10.1371/journal.pone.0155757)
Supplement: S4 Table — (DOCX) [file pone.0155757.s010.docx]

| **AMOVA test**  **Light harvesting phycobilisome alpha-subunit** | |  |  |
| --- | --- | --- | --- |
| Eastern_Tropical_Pacific-Galapagos-Indian_Ocean-Polynesia_Archipelagos-Sargasso-Tropical_South_Pacific | Among | Within | Total |
| SS | -86.0009 | 331.028 | 245.027 |
| df | 5 | 180 | 185 |
| MS | -17.2002 | 1.83904 |  |
| Fs: | -9.35278 |  |  |
| p-value: 0.938 | |  |  |
| **AMOVA test**  **Light harvesting phycobilisome beta-subunit** | |  |  |
| Atlantic-Eastern_Tropical_Pacific-Galapagos-Indian_Ocean-Polynesia_Archipelagos-Sargasso-Tropical_South_Pacific | Among | Within | Total |
| SS | 211.889 | 435.464 | 647.353 |
| df | 6 | 514 | 520 |
| MS | 35.3148 | 0.847207 |  |
| Fs: | 41.6837 |  |  |
| p-value: 0.001* |  |  |  |
| Atlantic-Eastern_Tropical_Pacific | Among | Within | Total |
| SS | 7.78036 | 22.5884 | 30.3688 |
| df | 1 | 52 | 53 |
| MS | 7.78036 | 0.434392 |  |
| Fs: | 17.9109 |  |  |
| p-value: 0.099 | |  |  |
| Atlantic-Galapagos | Among | Within | Total |
| SS | 77.3748 | 91.3371 | 168.712 |
| df | 1 | 123 | 124 |
| MS | 77.3748 | 0.742578 |  |
| Fs: | 104.198 |  |  |
| p-value: <0.001* |  |  |  |
| Atlantic-Indian_Ocean | Among | Within | Total |
| SS | 98.6758 | 119.981 | 218.657 |
| df | 1 | 221 | 222 |
| MS | 98.6758 | 0.5429 |  |
| Fs: | 181.757 |  |  |
| p-value: <0.001* | |  |  |
| Atlantic-Polynesia_Archipelagos | Among | Within | Total |
| SS | 72.4259 | 47.3428 | 119.769 |
| df | 1 | 63 | 64 |
| MS | 72.4259 | 0.751473 |  |
| Fs: | 96.3786 |  |  |
| p-value: <0.001* |  |  |  |
| Atlantic-Sargasso | Among | Within | Total |
| SS | 13.3552 | 91.8912 | 105.246 |
| df | 1 | 81 | 82 |
| MS | 13.3552 | 1.13446 |  |
| Fs: | 11.7723 |  |  |
| p-value: 0.265 | |  |  |
| Atlantic-Tropical_South_Pacific | Among | Within | Total |
| SS | -13.1709 | 134.563 | 121.392 |
| df | 1 | 114 | 115 |
| MS | -13.1709 | 1.18038 |  |
| Fs: | -11.1582 |  |  |
| p-value: 0.715 | |  |  |
| Eastern_Tropical_Pacific-Galapagos | Among | Within | Total |
| SS | 29.095 | 85.0299 | 114.125 |
| df | 1 | 119 | 120 |
| MS | 29.095 | 0.714537 |  |
| Fs: | 40.7187 |  |  |
| p-value: 0.098 | |  |  |
| Eastern_Tropical_Pacific-Indian_Ocean | Among | Within | Total |
| SS | -0.323026 | 113.674 | 113.351 |
| df | 1 | 217 | 218 |
| MS | -0.323026 | 0.523842 |  |
| Fs: | -0.616647 |  |  |
| p-value: 0.643 | |  |  |
| Eastern_Tropical_Pacific-Polynesia_Archipelagos | Among | Within | Total |
| SS | 8.31706 | 41.0357 | 49.3527 |
| df | 1 | 59 | 60 |
| MS | 8.31706 | 0.695519 |  |
| Fs: | 11.9581 |  |  |
| p-value: 0.137 | |  |  |
| Eastern_Tropical_Pacific-Sargasso | Among | Within | Total |
| SS | -9.15629 | 85.584 | 76.4277 |
| df | 1 | 77 | 78 |
| MS | -9.15629 | 1.11148 |  |
| Fs: | -8.23792 |  |  |
| p-value: 0.635 | |  |  |
| Eastern_Tropical_Pacific-Tropical_South_Pacific | Among | Within | Total |
| SS | 32.4178 | 128.256 | 160.674 |
| df | 1 | 110 | 111 |
| MS | 32.4178 | 1.16596 |  |
| Fs: | 27.8035 |  |  |
| p-value: 0.166 | |  |  |
| Galapagos-Indian_Ocean | Among | Within | Total |
| SS | 144.166 | 182.422 | 326.588 |
| df | 1 | 288 | 289 |
| MS | 144.166 | 0.633411 |  |
| Fs: | 227.602 |  |  |
| p-value: <0.001* |  |  |  |
| Galapagos-Polynesia_Archipelagos | Among | Within | Total |
| SS | -4.4957 | 109.784 | 105.289 |
| df | 1 | 130 | 131 |
| MS | -4.4957 | 0.844495 |  |
| Fs: | -5.32353 |  |  |
| p-value: 0.528 | |  |  |
| Galapagos-Sargasso | Among | Within | Total |
| SS | -11.7201 | 154.333 | 142.613 |
| df | 1 | 148 | 149 |
| MS | -11.7201 | 1.04279 |  |
| Fs: | -11.2392 |  |  |
| p-value: 0.722 | |  |  |
| Galapagos-Tropical_South_Pacific | Among | Within | Total |
| SS | 73.9441 | 197.005 | 270.949 |
| df | 1 | 181 | 182 |
| MS | 73.9441 | 1.08842 |  |
| Fs: | 67.9369 |  |  |
| p-value: 0.013 |  |  |  |
| Indian_Ocean-Polynesia_Archipelagos | Among | Within | Total |
| SS | 77.2947 | 138.428 | 215.723 |
| df | 1 | 228 | 229 |
| MS | 77.2947 | 0.607141 |  |
| Fs: | 127.309 |  |  |
| p-value: <0.001* |  |  |  |
| Indian_Ocean-Sargasso | Among | Within | Total |
| SS | -16.0743 | 182.976 | 166.902 |
| df | 1 | 246 | 247 |
| MS | -16.0743 | 0.743807 |  |
| Fs: | -21.6108 |  |  |
| p-value: 0.891 | |  |  |
| Indian_Ocean-Tropical_South_Pacific | Among | Within | Total |
| SS | 16.5193 | 225.648 | 242.168 |
| df | 1 | 279 | 280 |
| MS | 16.5193 | 0.808776 |  |
|  |  |  |  |
| Fs: | 20.4251 |  |  |
| p-value: 0.237 | |  |  |
| Polynesia_Archipelagos-Sargasso | Among | Within | Total |
| SS | -14.1114 | 110.338 | 96.227 |
| df | 1 | 88 | 89 |
| MS | -14.1114 | 1.25385 |  |
| Fs: | -11.2545 |  |  |
| p-value: 0.746 | |  |  |
| Polynesia_Archipelagos-Tropical_South_Pacific | Among | Within | Total |
| SS | 27.3403 | 153.01 | 180.351 |
| df | 1 | 121 | 122 |
| MS | 27.3403 | 1.26455 |  |
| Fs: | 21.6206 |  |  |
| p-value: 0.24 | |  |  |
| Sargasso-Tropical_South_Pacific | Among | Within | Total |
| SS | -45.1788 | 197.559 | 152.38 |
| df | 1 | 139 | 140 |
| MS | -45.1788 | 1.42129 |  |
| Fs: | -31.7872 |  |  |
| p-value: 0.961 | |  |  |
| **AMOVA test**  **Chlorophyll-binding peptides (Pcb/IsiA)** | |  |  |
| Atlantic-Eastern_Tropical_Pacific-Galapagos-Indian_Ocean-Polynesia_Archipelagos-Sargasso-Tropical_South_Pacific | Among | Within | Total |
| SS | -414.815 | 2256.44 | 1841.63 |
| df | 6 | 816 | 822 |
| MS | -69.1359 | 2.76525 |  |
| Fs: | -25.0017 |  |  |
| p-value: 0.995 | |  |  |
